# Integrated Metabolomics and Multilevel Validation of the Anti-Inflammatory and Skin-Soothing Effects of Bioactive Components from *Prinsepia utilis* Seeds

**DOI:** 10.3390/ijms27177951

**Published:** 2026-09-07

**Authors:** Ruyi He, Changran Li, Xiaoxue Mao, Chao Huang, Mengjiao Yang, Jianqin Li, Xiaoli Wu, Lixin Yang

**Affiliations:** 1 Key Laboratory of Economic Plants and Biotechnology, Kunming Institute of Botany, Chinese Academy of Sciences, Kunming 650201, China; 2 School of Ethnic Medicine, Yunnan Minzu University, Kunming 650201, China; 3 School of Economics and Management, Southwest Forestry University, Kunming 650201, China; 4 Institute of Yangming and Guizhou Studies, Guiyang University, Guiyang 550005, China; 5 Bio-Innovation Center of DR PLANT, Kunming Institute of Botany, Chinese Academy of Sciences, Kunming 650201, China

**Keywords:** *Prinsepia utilis*, untargeted metabolomics, bioactive metabolites, molecular dynamics, 3D epidermal model, zebrafish, anti-inflammatory activity, skin-soothing efficacy, AKT/MAPK signaling pathways

## Abstract

*Prinsepia utilis* Royle seeds are traditionally used in Northwestern Yunnan to relieve skin inflammation and irritation. However, its active constituents and potential molecular mechanisms remain unclear. To investigate the bioactive constituents and anti-inflammatory and skin-soothing effects of *P. utilis* seed-derived extracts, we characterized their chemical profiles and investigated the potential molecular mechanisms through phytochemical, computational, and complementary biological approaches, as well as in vitro and in vivo tests in this study. The results of this study show that the 75% ethanol eluate (QC04) of *P. utilis* seeds exhibited the strongest inhibitory effect on lipopolysaccharide-induced nitric oxide production in RAW 264.7 macrophages, with an IC_50_ value of 239.21 ± 6.70 μg/mL. Subsequently, untargeted metabolomics identified 1039 metabolites, and 20 representative compounds were selected for downstream analysis. Furthermore, network pharmacology analysis identified AKT1, MAPK1, MAPK8, and MAPK14 as core targets, which are mainly involved in the TNF and MAPK signaling pathways. Molecular docking confirmed favorable binding interactions between representative metabolites and these core proteins; meanwhile, a 100 ns molecular dynamics simulation verified the conformational stability of the euscaphic acid–AKT1 complex. Moreover, in the UVB-induced 3D epidermal model (EpiKutis^®^), QC04 treatment reduced IL-6 and PGE_2_ secretion and downregulated TNF-α and NF-κB p65 expression. In vivo zebrafish assays further demonstrated that QC04 inhibited copper sulfate-induced neutrophil recruitment and histamine-induced vasodilation. Overall, these findings link the metabolites identified in the activity-enriched QC04 fraction with predicted molecular targets and anti-inflammatory and skin-soothing effects, providing experimental evidence for the biological potential of *P. utilis* seed-derived preparations and supporting further investigation of QC04 as a potential skin-soothing active fraction.

## 1. Introduction

*Prinsepia utilis* Royle (Rosaceae) is a perennial deciduous shrub native to the Himalayan region and naturally distributed at elevations of 1600–3000 m across India, Nepal, Bangladesh, and Southwestern China [1]. Wild populations of this species are primarily concentrated across Yunnan, Sichuan, Guizhou, and the Xizang Autonomous Region [2], with Northwestern Yunnan—particularly the prefectures of Lijiang, Dali, and Diqing—recognized as one of its core distribution centers. Generally, *P. utilis* preferentially grows on hillsides, riverbanks, valleys, wastelands, and shrublands at elevations of 2100–2700 m [3]. Traditionally, local ethnic communities have utilized the young shoots, leaves, stems, and fruits of *P. utilis*, including cold-pressed seed oil, as both food resources and traditional herbal medicines [4]. Traditional medical records and pharmacological studies have shown that *P. utilis* seed oil has long been applied topically to alleviate inflammatory skin disorders, serve as infant skincare oil, promote wound healing, and relieve musculoskeletal pain and rheumatic diseases [5,6,7]. Additionally, recent studies further suggest its potential for alleviating cutaneous erythema, dryness, and pruritus, highlighting its promise for dermatological applications [8,9].

*P. utilis* seeds are rich in a wide range of bioactive constituents, including flavonoids, terpenoids, phenolic acids, lignans, polysaccharides, and unsaturated fatty acids [9,10]. Previous phytochemical investigations have identified numerous secondary metabolites from different tissues of *P. utilis*, underscoring the remarkable chemical diversity of this species [11,12]. In particular, the seed oil contains abundant oleic and linoleic acids, which are well-documented to promote epidermal barrier repair and modulate cutaneous inflammatory responses [13,14]. Consistently, pharmacological studies have demonstrated that *P. utilis* seed extracts and seed oil possess antioxidant [15], anti-inflammatory and analgesic [7], and antibacterial activities [9], while flavonoid-rich fractions derived from seed residues also exhibit anti-senescent and skin-protective effects in animal models [16]. These previous findings indicate that the biological activities of *P. utilis* are closely associated with its chemically diverse seed-derived constituents, while also suggesting that different seed-derived preparations may exert distinct biological effects. Overall, these findings indicate that *P. utilis* seeds constitute a promising source of natural bioactive metabolites for dermatological applications.

Given the reported dermatological uses of *P. utilis* seed-derived preparations, inflammation was selected as a relevant biological endpoint to evaluate their potential anti-inflammatory activity. Inflammation is essential for host defense and tissue repair, but persistent or dysregulated inflammation is associated with various inflammatory diseases, including inflammatory dermatoses, arthritis, and cardiovascular diseases [7,17]. Therefore, screening safe and efficient anti-inflammatory active substances from natural medicinal plants has become a major focus of modern drug discovery [18]. Natural products, in particular, have attracted increasing attention owing to their exceptional structural diversity and multi-target pharmacological activities. Indeed, prior studies have preliminarily verified the anti-inflammatory activity of extracts from the leaves, fruits and roots of *P. utilis* [9,19]. Despite increasing interest in *P. utilis* seeds, several important knowledge gaps remain. Specifically, comprehensive studies systematically elucidating its multi-target and multi-pathway anti-inflammatory properties using advanced in vitro and in vivo models are still limited. Furthermore, the chemical profiling and functional interpretation of its characteristic bioactive metabolites remain insufficient and unsystematic. More importantly, the underlying molecular mechanisms by which *P. utilis* Royle seed extract regulates inflammatory responses and alleviates UVB-induced skin damage warrant further investigation.

To address these knowledge gaps, an activity-guided extract fraction (QC04) was prepared from *P. utilis* seeds based on its inhibitory activity against nitric oxide (NO) production. The present study aimed to identify the most active seed fraction, characterize its major bioactive metabolites, and investigate its potential anti-inflammatory mechanisms and skin-soothing effects through complementary chemical, computational, and biological approaches. Subsequently, untargeted metabolomics was employed to comprehensively characterize its chemical composition. Next, network pharmacology, molecular docking, and molecular dynamics simulation were integrated to identify key anti-inflammatory targets and predict interactions between representative metabolites and core proteins. Then, the predicted molecular network was evaluated using a UVB-induced in vitro reconstructed three-dimensional (3D) human epidermal model (EpiKutis^®^) by measuring TNF-α, NF-κB p65, IL-6, and PGE_2_, while zebrafish embryo models were employed to further assess functional inflammatory responses through neutrophil recruitment and histamine-induced vasodilation assays [20,21]. Overall, this study integrates phytochemical characterization, computational prediction, and biological evaluation to investigate the potential anti-inflammatory mechanisms and skin-protective effects of *P. utilis* seed extract. The findings are expected to provide experimental evidence for the biological potential of *P. utilis* seed-derived fractions and support further investigation of their skin-soothing applications.

## 2. Results

### 2.1. Preparation and Fractionation of the Crude Extract

Ultrasound-assisted extraction of 3.0 kg of *P. utilis* seeds with 95% ethanol yielded 208 g of crude ethanol extract, corresponding to a yield of 6.93%. Subsequent D101 macroporous resin fractionation of 200 g of the crude extract yielded 64.0 g of the water fraction (QC01, 32.0%), 18.6 g of 25% ethanol fraction (QC02, 9.3%), 17.7 g of 50% ethanol fraction (QC03, 8.85%), and 13.0 g of 75% ethanol fraction (QC04, 6.5%), respectively. With increasing ethanol concentration, the fractions became progressively darker and less soluble in water, indicating the gradual enrichment of less polar constituents in the later eluates.

### 2.2. Inhibitory Effects of the Fractions on NO Production

**Figure 1:**
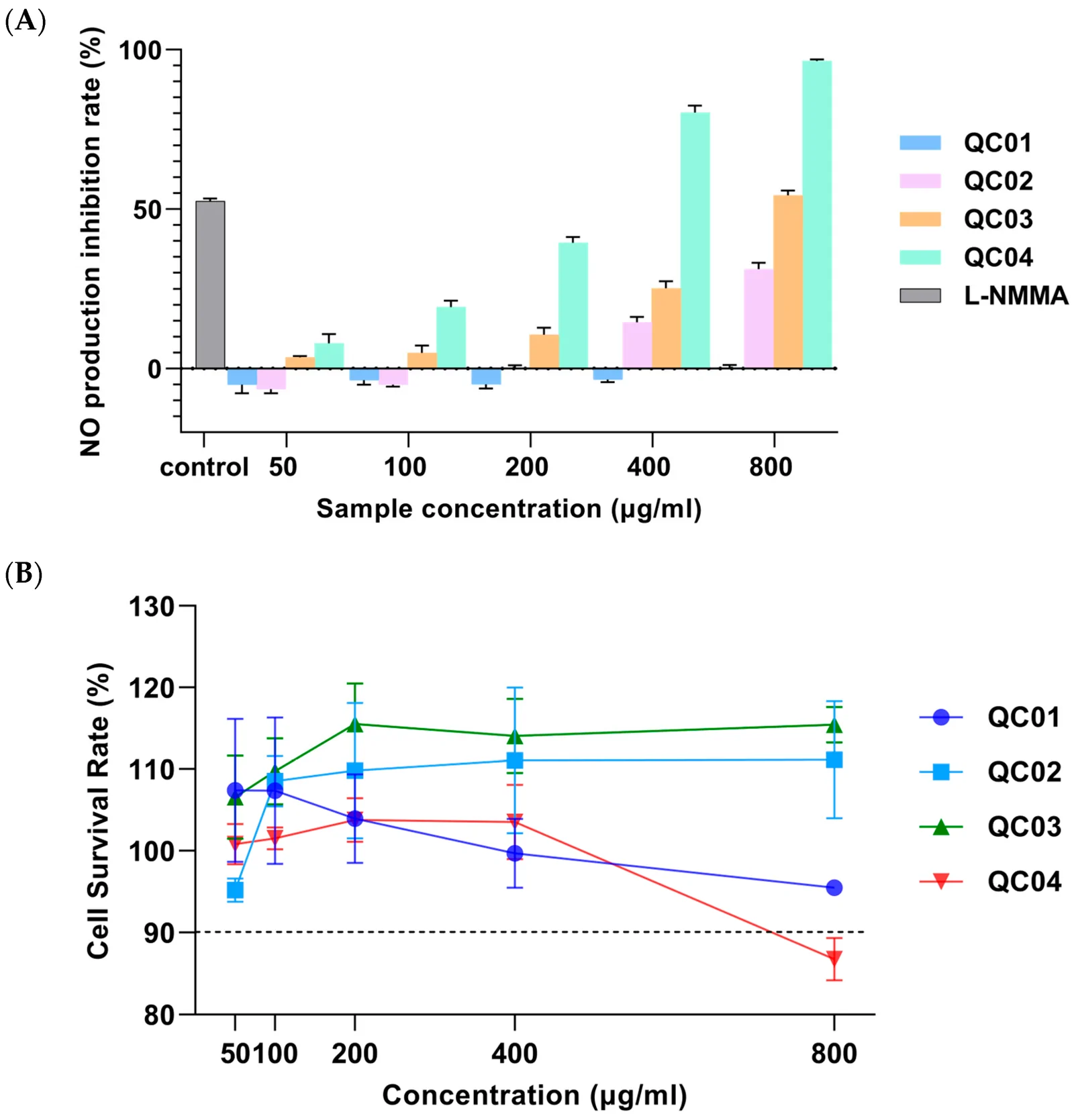
Inhibitory effects of QC01-QC04 on NO production and cell viability in LPS-stimulated RAW 264.7 macrophages. (**A**) Dose–response curves of NO production inhibition. (**B**) Corresponding cell viability curves; the dashed line at 90% viability marks the non-cytotoxic threshold. L-NMMA served as the positive control. Data are shown as mean ± SD from three technical replicates.

The anti-inflammatory activity of QC01–QC04 was evaluated by measuring NO production in LPS-stimulated RAW 264.7 macrophages, with cell viability assayed in parallel to exclude cytotoxic interference (Figure 1). QC04 exhibited the strongest concentration-dependent NO inhibitory activity, with an IC_50_ of 239.21 ± 6.70 μg/mL (95% CI: 231.62–246.80 μg/mL). At 50–400 μg/mL, cell viability remained above 90% (the predefined non-cytotoxic range), and the inhibition rate reached 80.31% at 400 μg/mL. At 800 μg/mL, cell viability dropped to 86.7%, below the 90% threshold; this concentration was excluded from IC_50_ calculation as its effect was confounded by cytotoxicity. QC03 showed moderate activity (IC_50_ = 721.84 ± 26.44 μg/mL), whereas QC01 and QC02 displayed only weak inhibitory effects. Given its superior potency within the non-cytotoxic range, QC04 was selected for subsequent chemical and mechanistic investigations.

### 2.3. Metabolite Profiling and Selection of Representative Compounds

**Figure 2:**
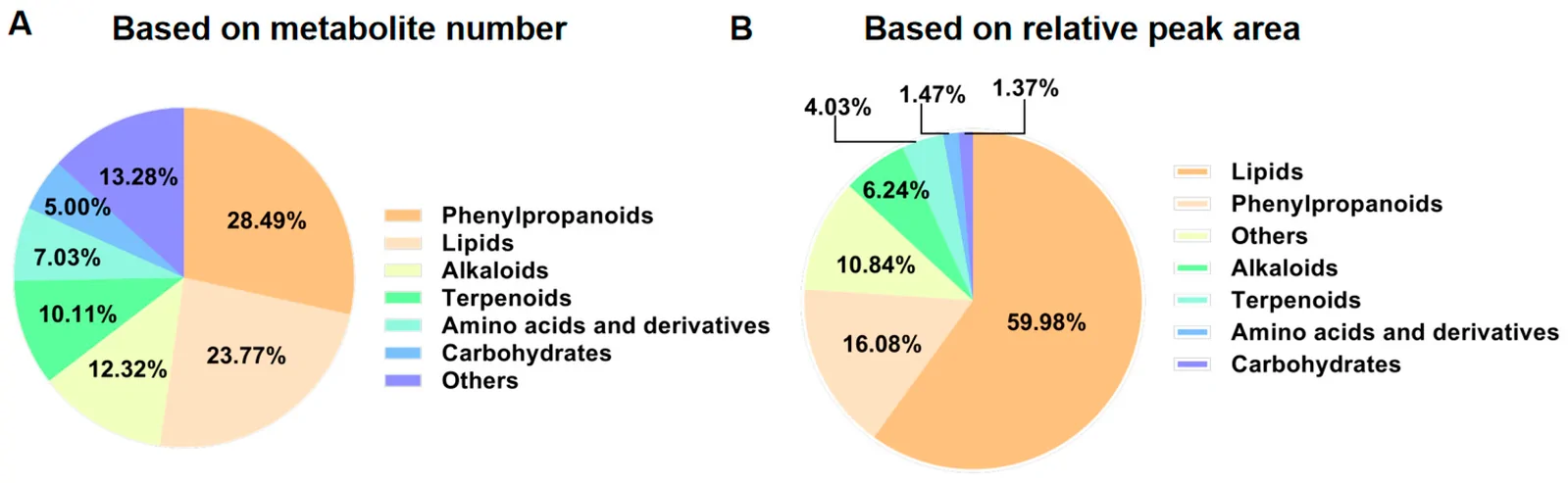
Chemical class distribution of metabolites identified in the QC04 fraction. (**A**) Percentage of metabolites in each chemical class based on the total number of identified metabolites (*n* = 1039). (**B**) Relative peak-area abundance of each chemical class based on the summed mean peak areas of all identified metabolites.

Untargeted metabolomic analysis of QC04 identified a total of 1039 metabolites (Supplementary File S1), all of which were assigned MSI Level 1 confidence according to the Metabolomics Standards Initiative criteria [22]. Complete annotation details for all 1039 identified metabolites—including precursor m/z, mass error, retention time, ionization mode, adduct type, molecular formula, major MS/MS fragment ions, library match score, and assigned confidence level—are summarized in Supplementary Table S1 to enable full traceability of the identification results. As further evidence of the reliability of the annotations, Supplementary File S2 provides experimental MS/MS spectra for all 20 representative metabolites selected for network pharmacology analysis—including the six core compounds used for molecular docking—along with mirror comparisons to authentic standard reference spectra. Based on their structural characteristics, we found that these metabolites were classified into seven major chemical categories (Figure 2). Phenylpropanoids represented the largest proportion (28.49%), followed by lipids (23.77%), alkaloids (12.32%), terpenoids (10.11%), amino acids and derivatives (7.03%), carbohydrates (5.00%), and other metabolites (13.28%), whereas lipids showed the highest relative peak-area abundance (59.98%).

Based primarily on MS/MS matching scores (≥3.95), relative signal abundance, and chemical-class representation, 20 representative metabolites were selected for subsequent network pharmacology analysis (Table 1). These compounds were mainly phenylpropanoids and terpenoids, including flavonoids, phenolic acids, triterpenoids, and saponins, indicating that these metabolite classes may contribute to the anti-inflammatory activity of QC04 [23,24].

### 2.4. Bioinformatics Analysis, Docking and Molecular Dynamics Simulation

#### 2.4.1. Identification and Enrichment Analysis of Candidate Anti-Inflammatory Targets

**Figure 3:**
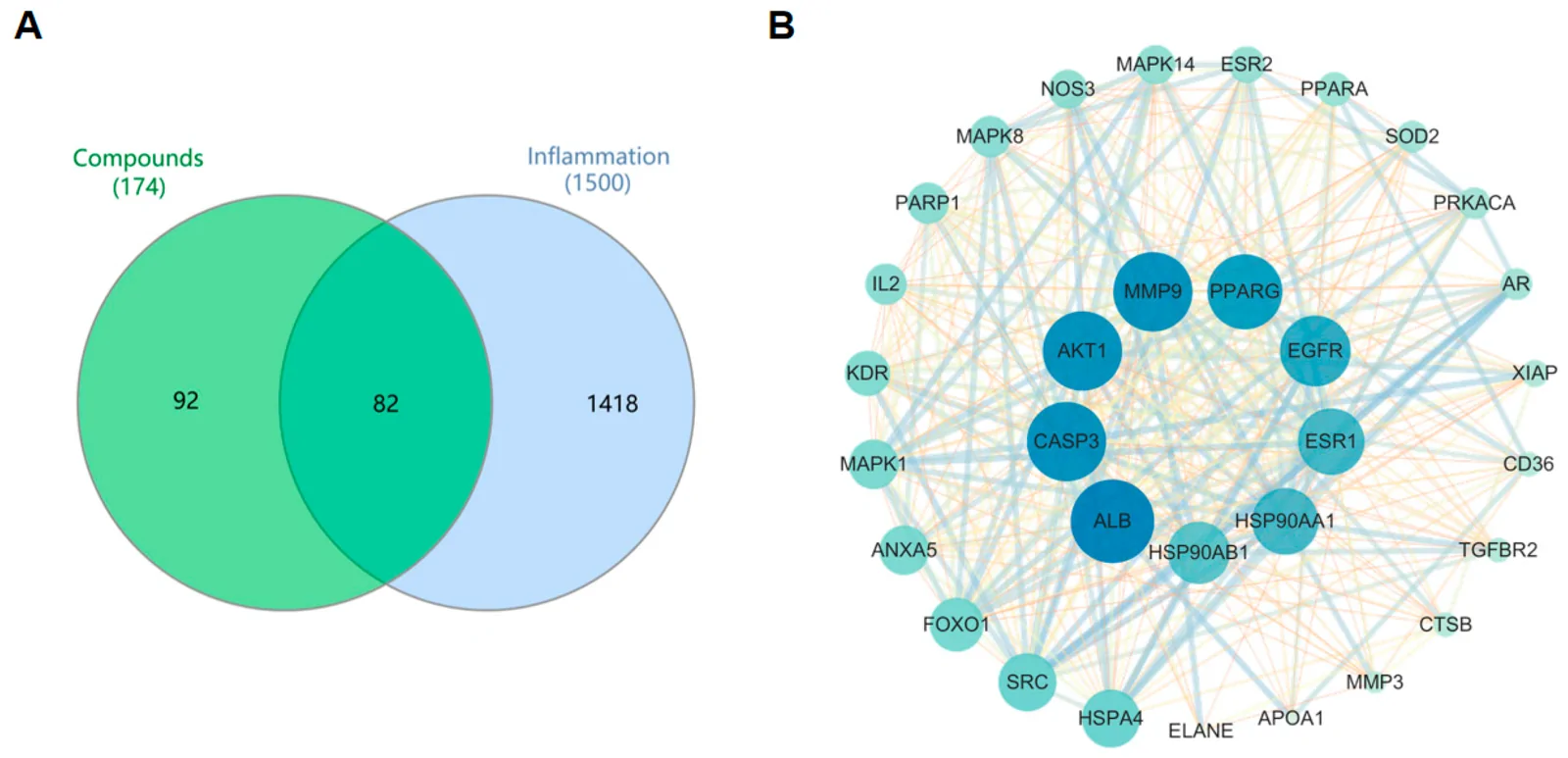
Identification of candidate anti-inflammatory targets of QC04. (**A**) Venn diagram showing the overlap between metabolite-related and inflammation-associated targets. (**B**) Protein–protein interaction network of the core targets. Node size and color intensity indicate the degree of connectivity.

Network pharmacology analysis was performed using the 20 representative metabolites selected from QC04 [18]. A total of 174 metabolite-related targets and 1500 inflammation-associated targets were retrieved, and 82 overlapping genes were identified as potential anti-inflammatory targets of QC04 (Figure 3A) in this study.

The 82 overlapping targets were further analyzed using a protein–protein interaction network. Several highly connected genes (Supplementary Table S2), including ALB, MMP9, AKT1, CASP3, ESR1, PPARG, EGFR, and HSP90AA1, were identified as hub targets (Figure 3B). Among them, AKT1, MAPK1, MAPK8, and MAPK14 were selected for downstream structural validation as core druggable kinases of inflammatory signaling cascades, and they are enriched in the top-ranked KEGG pathways identified in this study [25].

**Figure 4:**
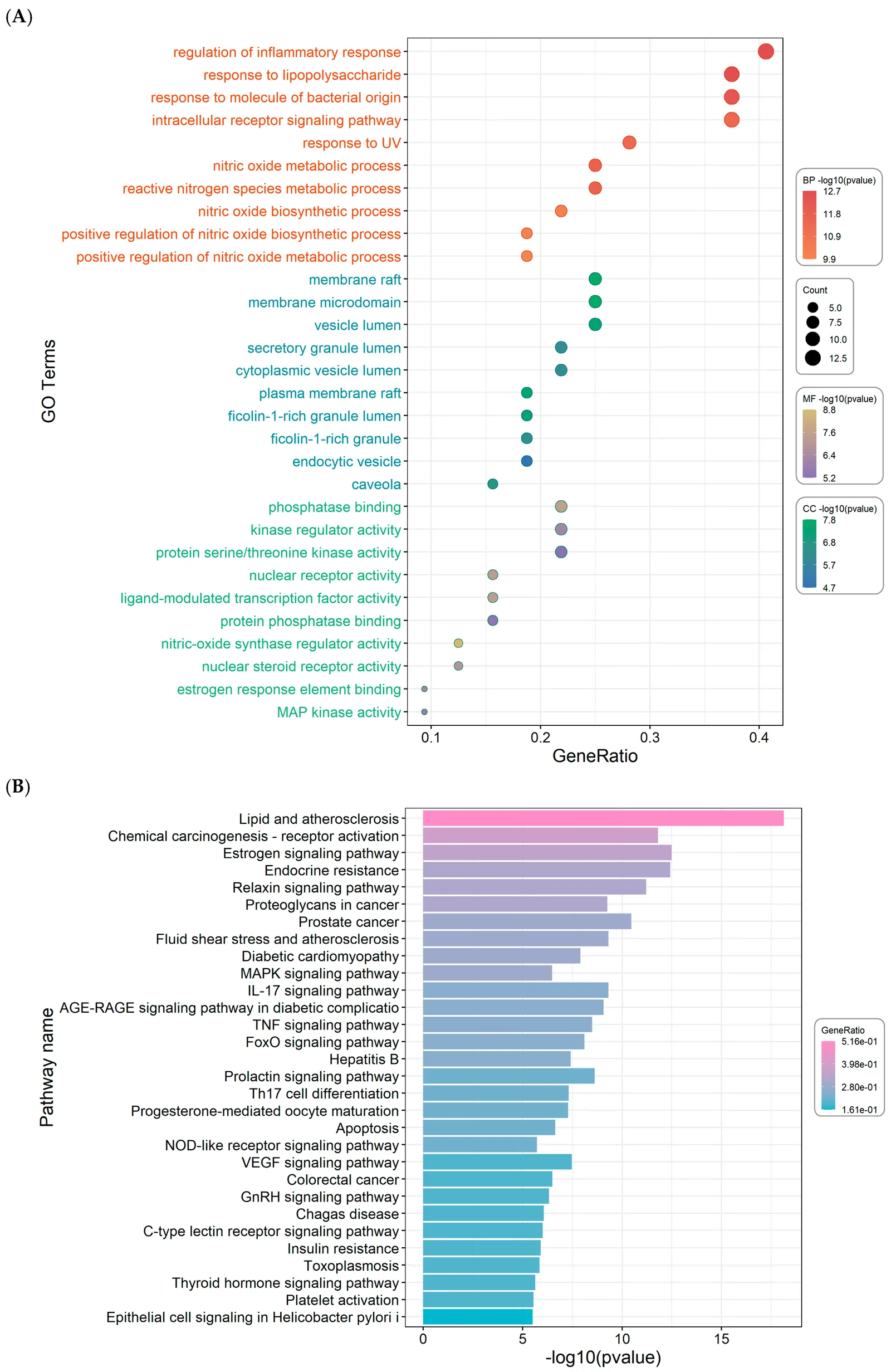
Functional enrichment analysis of the overlapping targets. (**A**) Gene Ontology enrichment analysis, including biological process, cellular component, and molecular function categories. Dot size represents the number of enriched genes, and dot color indicates statistical significance. (**B**) KEGG pathway enrichment analysis. Bar length represents statistical significance, and bar color indicates the gene ratio.

Gene Ontology (GO) enrichment analysis showed that the candidate targets were mainly associated with the regulation of inflammatory responses, responses to lipopolysaccharide and ultraviolet radiation, and nitric oxide metabolism (Figure 4A). The enriched cellular components included membrane rafts, vesicle lumens, and caveolae, whereas the principal molecular functions involved protein kinase regulation, serine/threonine kinase activity, and MAP kinase activity [26].

KEGG enrichment analysis indicated that the candidate targets were primarily involved in the TNF, MAPK, IL-17, and FoxO signaling pathways (Figure 4B). These pathways are closely associated with inflammatory signaling and UVB-induced epidermal injury [26,27]. Based on the network topology and enrichment results, TNF-α, NF-κB, IL-6, and PGE_2_ were selected as downstream indicators for subsequent biological evaluations. Overall, these findings suggest that QC04 may exert anti-inflammatory effects through multiple kinase-associated targets and inflammatory signaling pathways [28].

#### 2.4.2. Molecular Docking Results

**Figure 5:**
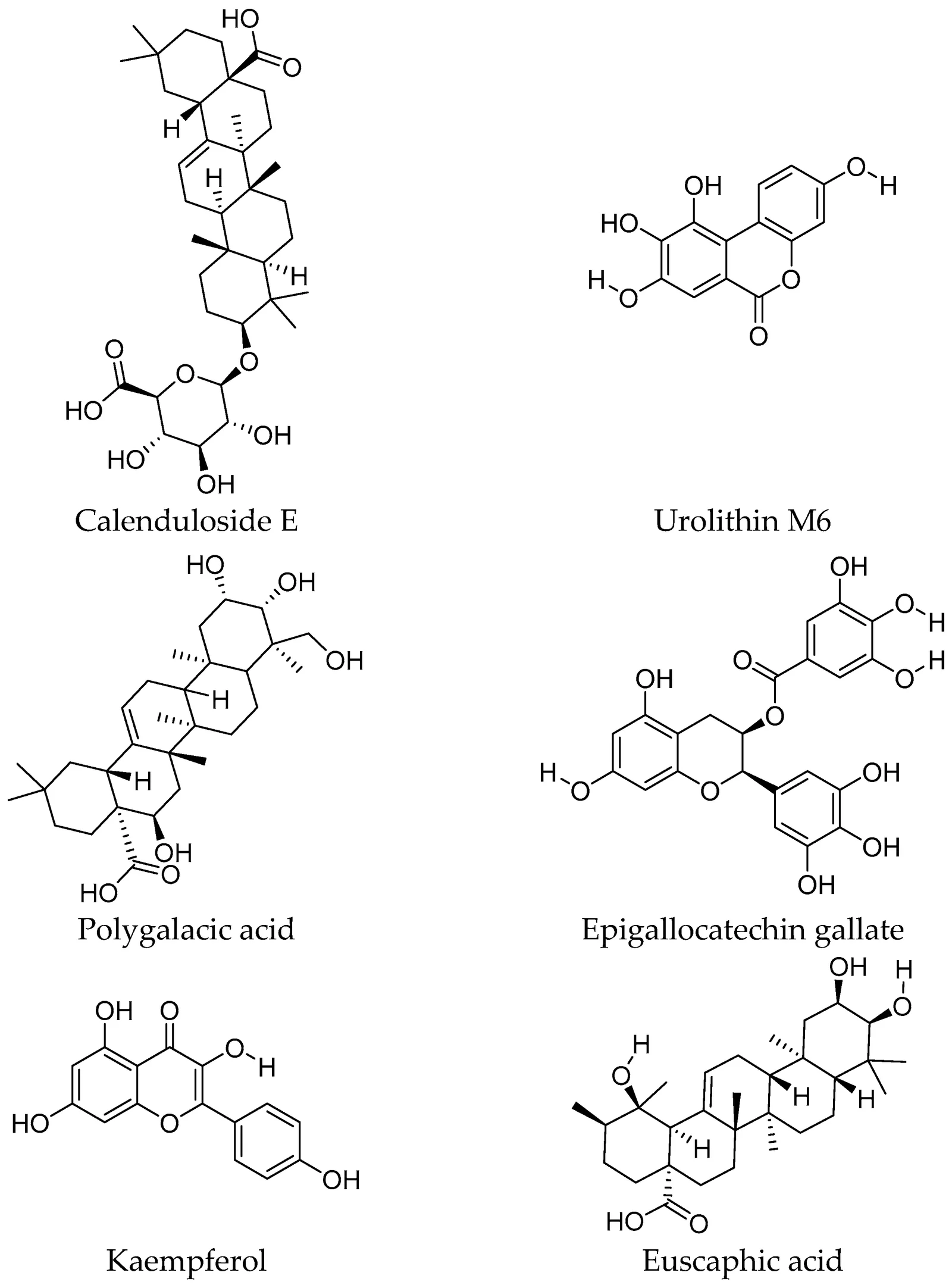
Chemical structures of the six representative metabolites selected for molecular docking.

Six representative metabolites from the present study—Calenduloside E, Urolithin M6, polygalacic acid, epigallocatechin gallate (EGCG), kaempferol, and euscaphic acid—were selected for molecular docking based on their relative abundance and reported anti-inflammatory potential (Figure 5). These compounds were docked against four hub targets identified by network analysis: AKT1, MAPK1, MAPK8, and MAPK14 [23,24].

**Figure 6:**
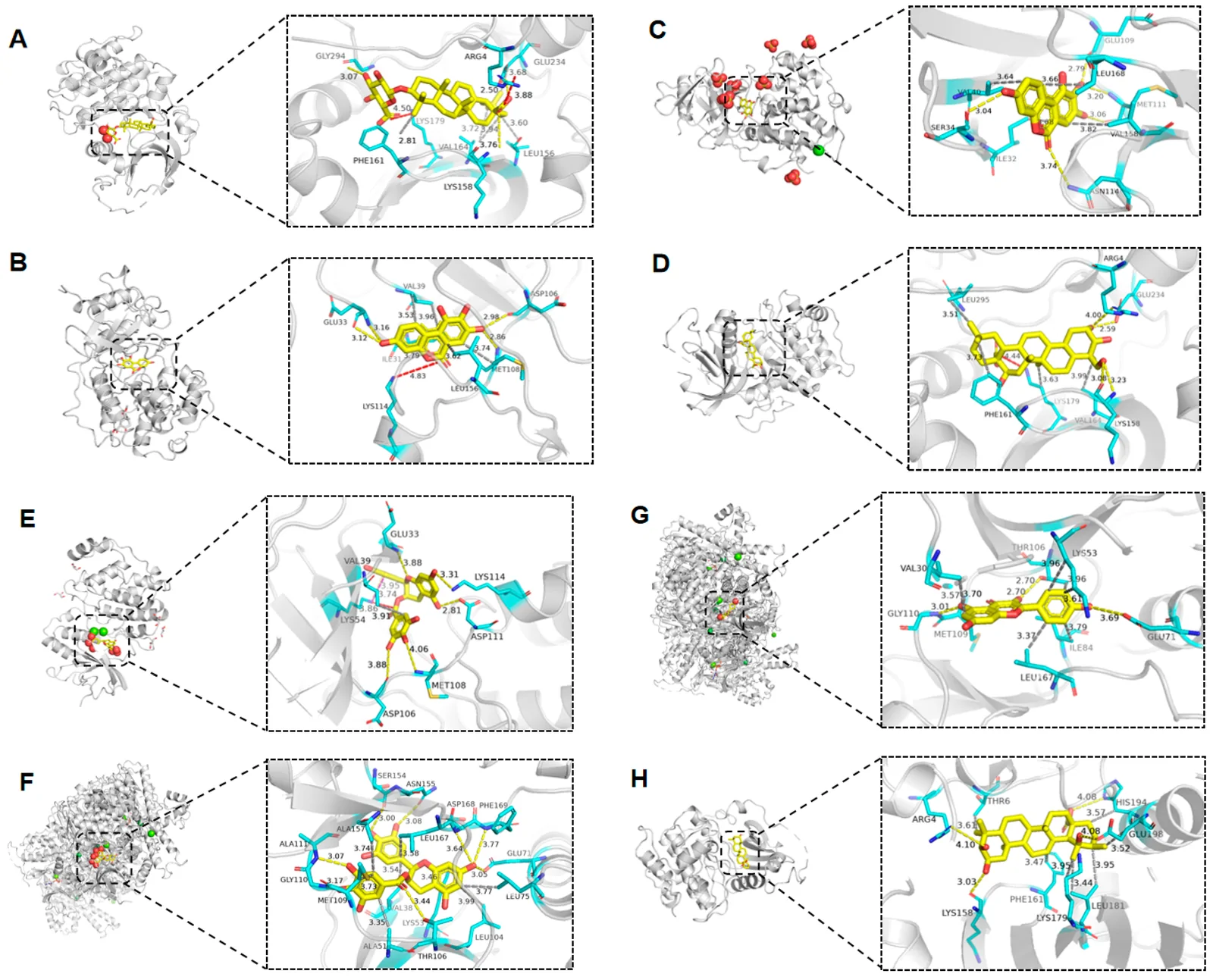
Representative binding modes of six QC04 metabolites with four core targets. (**A**) Calenduloside E-AKT1 (PDB ID: 3QKK), −9.7 kcal/mol; (**B**) Urolithin M6-MAPK1 (PDB ID: 8PT1), −9.8 kcal/mol; (**C**) Urolithin M6-MAPK8 (PDB ID: 3V3V), −8.9 kcal/mol; (**D**) polygalacic acid–AKT1 (PDB ID: 3QKK), −9.3 kcal/mol; (**E**) EGCG-MAPK1 (PDB ID: 5V60), −9.0 kcal/mol; (**F**) EGCG-MAPK14 (PDB ID: 5NZZ), −10.2 kcal/mol; (**G**) kaempferol–MAPK14 (PDB ID: 5NZZ), −8.6 kcal/mol; and (**H**) euscaphic acid–AKT1 (PDB ID: 3QKK), −9.9 kcal/mol.

All evaluated complexes showed favorable predicted binding energies (−10.2 to −8.6 kcal/mol), indicating good structural complementarity [29]. The full docking score matrix is provided in Supplementary Table S3. Among them, the EGCG-MAPK14 complex exhibited the lowest binding energy (−10.2 kcal/mol), followed by euscaphic acid–AKT1 (−9.9 kcal/mol), Urolithin M6-MAPK1 (−9.8 kcal/mol), and Calenduloside E-AKT1 (−9.7 kcal/mol). Eight representative complexes were selected for visualization (Figure 6).

The ligands occupied the binding pockets of their respective targets and were stabilized mainly by hydrogen bonds and hydrophobic interactions, with additional salt bridges or π-cation interactions observed in several complexes. EGCG formed extensive hydrogen-bonding and hydrophobic interactions with MAPK14, whereas euscaphic acid interacted with AKT1 through hydrogen bonds involving LYS158, GLU198, HIS194, and ARG4, together with multiple hydrophobic contacts. Similar interactions were observed for Calenduloside E and polygalacic acid with AKT1, Urolithin M6 with MAPK1 and MAPK8, and kaempferol with MAPK14. These results support the predicted interactions of QC04 metabolites with AKT1 and MAPK-family proteins.

#### 2.4.3. Molecular Dynamics Simulation

**Figure 7:**
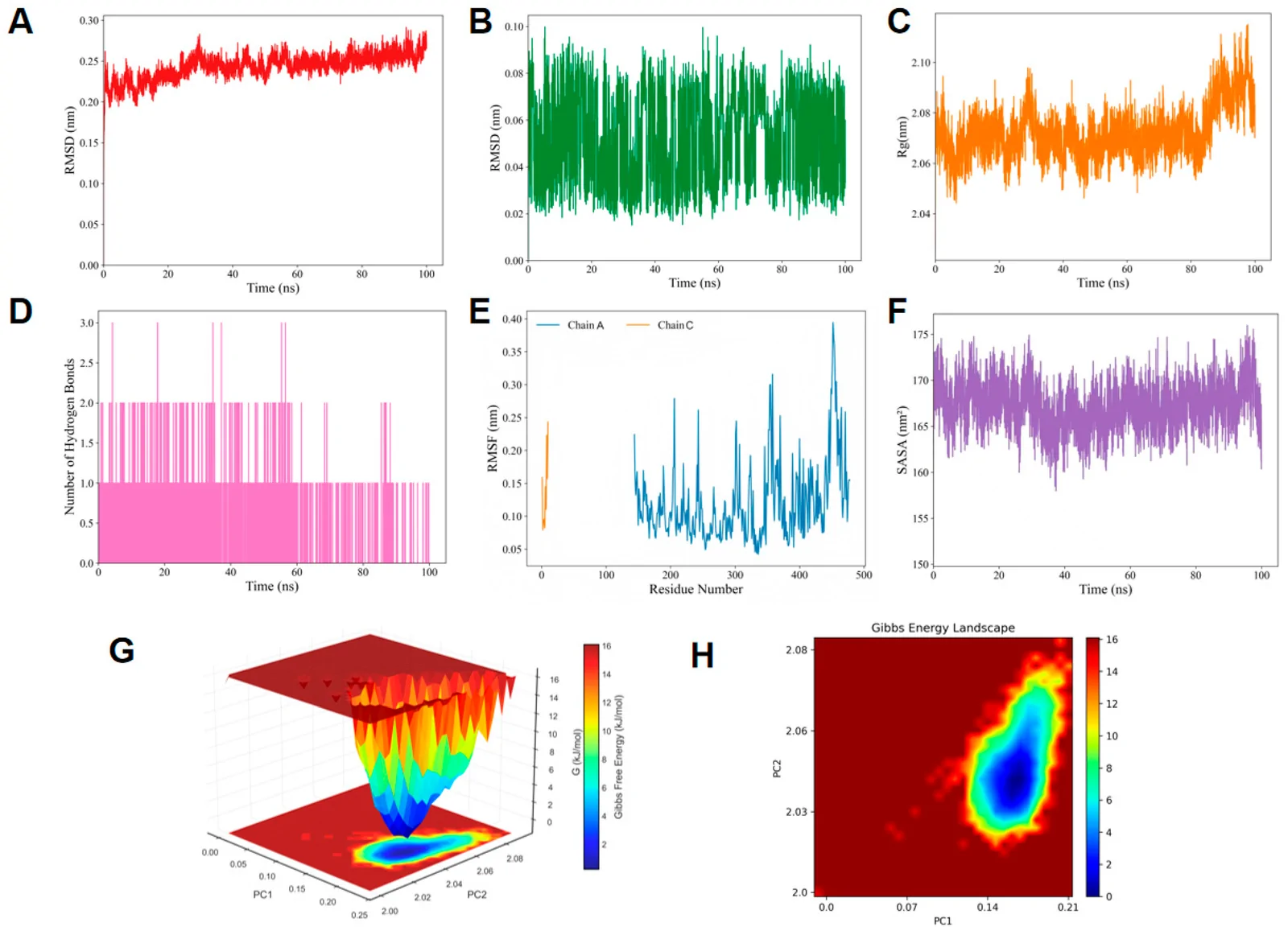
Molecular dynamics simulation of the euscaphic acid–AKT1 complex over 100 ns. (**A**) RMSD of the AKT1 protein backbone; (**B**) RMSD of euscaphic acid; (**C**) radius of gyration of the complex; (**D**) number of protein–ligand hydrogen bonds; (**E**) residue-level Cα RMSF of AKT1; (**F**) solvent-accessible surface area; (**G**) three-dimensional Gibbs free-energy landscape; and (**H**) the corresponding two-dimensional contour map.

The stability of the euscaphic acid–AKT1 complex was further assessed by a 100 ns molecular dynamics simulation. The RMSD of the AKT1 backbone reached equilibrium after approximately 25 ns and remained stable at around 0.25 nm. The ligand RMSD fluctuated near 0.05 nm without appreciable drift, indicating that euscaphic acid remained stably positioned within the AKT1 binding pocket throughout the simulation (Figure 7A,B).

The radius of gyration remained approximately 2.07 nm, suggesting that the complex maintained a compact conformation during the simulation (Figure 7C). The solvent-accessible surface area fluctuated slightly between 165 and 170 nm^2^, indicating that ligand binding did not induce substantial changes in the overall protein structure (Figure 7D). Between zero and three hydrogen bonds were formed between euscaphic acid and AKT1 during the simulation (Figure 7E). RMSF analysis showed relatively low fluctuations for most residues, with greater flexibility mainly observed in loop regions and at the C-terminus (Figure 7F).

The Gibbs free-energy landscape exhibited a predominant low-energy basin, indicating that the complex adopted a relatively stable conformational state. Consistent results from two independent 100 ns replicate simulations supported the structural stability of the euscaphic acid–AKT1 complex (Figure 7G,H; reproducibility data in Supplementary File S3) [30].

### 2.5. Anti-Inflammatory Effects of QC04 in the 3D Epidermal Model (EpiKutis^®^)

#### 2.5.1. Cytotoxicity of QC04 in Human Keratinocytes

**Figure 8:**
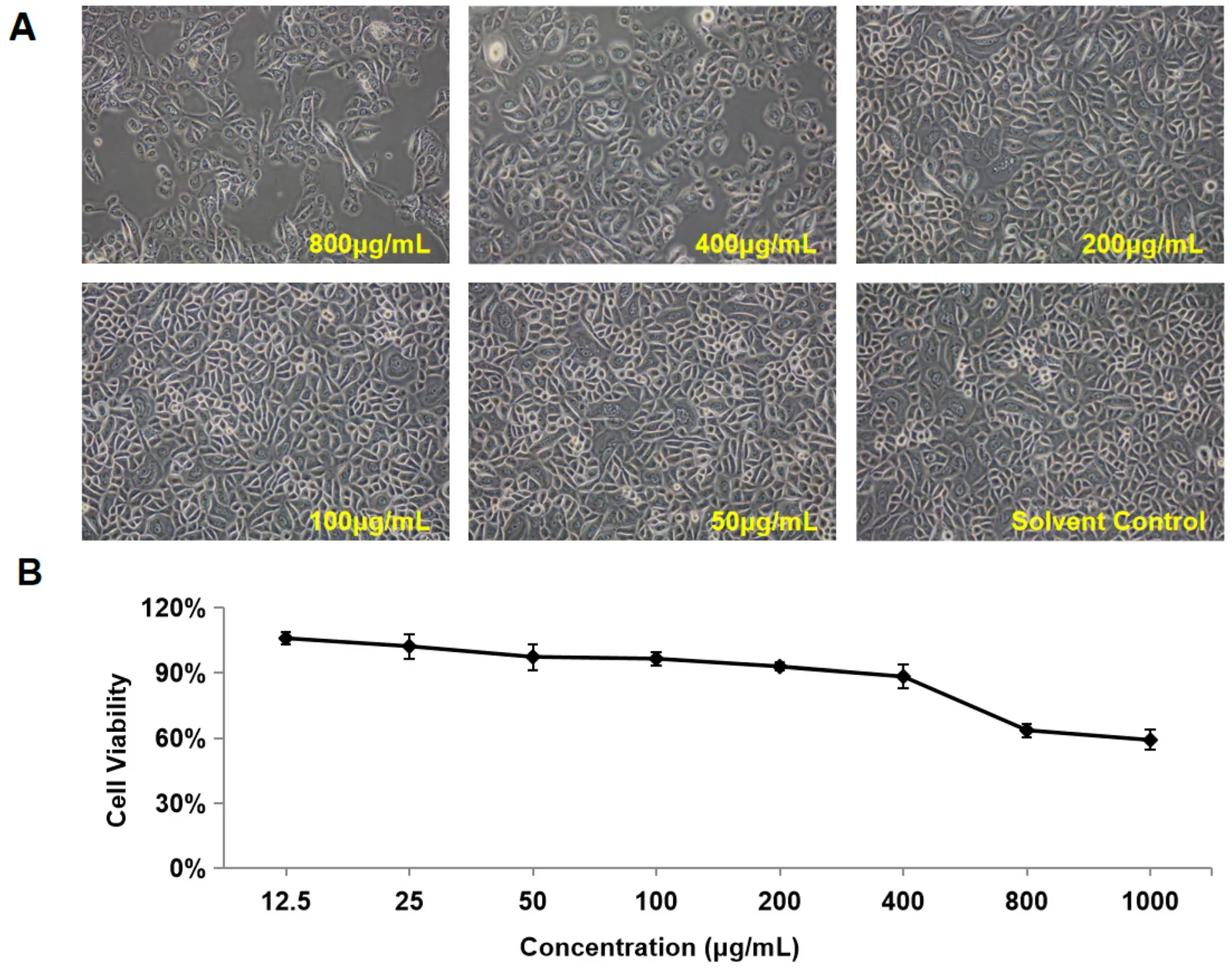
Cytotoxicity of QC04 in human keratinocytes. (**A**) Representative microscopic images of cells treated with QC04 at 50, 100, 200, 400, and 800 μg/mL, together with the vehicle control. (**B**) Cell viability after 24 h of treatment with QC04 at 12.5–1000 μg/mL, as determined by the MTT assay. Data are presented as the mean ± SD.

The cytotoxicity of QC04 was evaluated in human keratinocytes using the MTT assay [31]. Cell viability remained above 90% following treatment with 12.5–200 μg/mL QC04, and no obvious morphological changes were observed within this concentration range (Figure 8). At 400 μg/mL, cell viability decreased to 88.42%, while exposure to 800 and 1000 μg/mL reduced viability to 63.67% and 59.14%, respectively. Based on these results, 200 μg/mL was selected as the highest non-cytotoxic concentration for subsequent experiments.

#### 2.5.2. Effects of QC04 on UVB-Induced IL-6 and PEG_2_ Production

**Figure 9:**
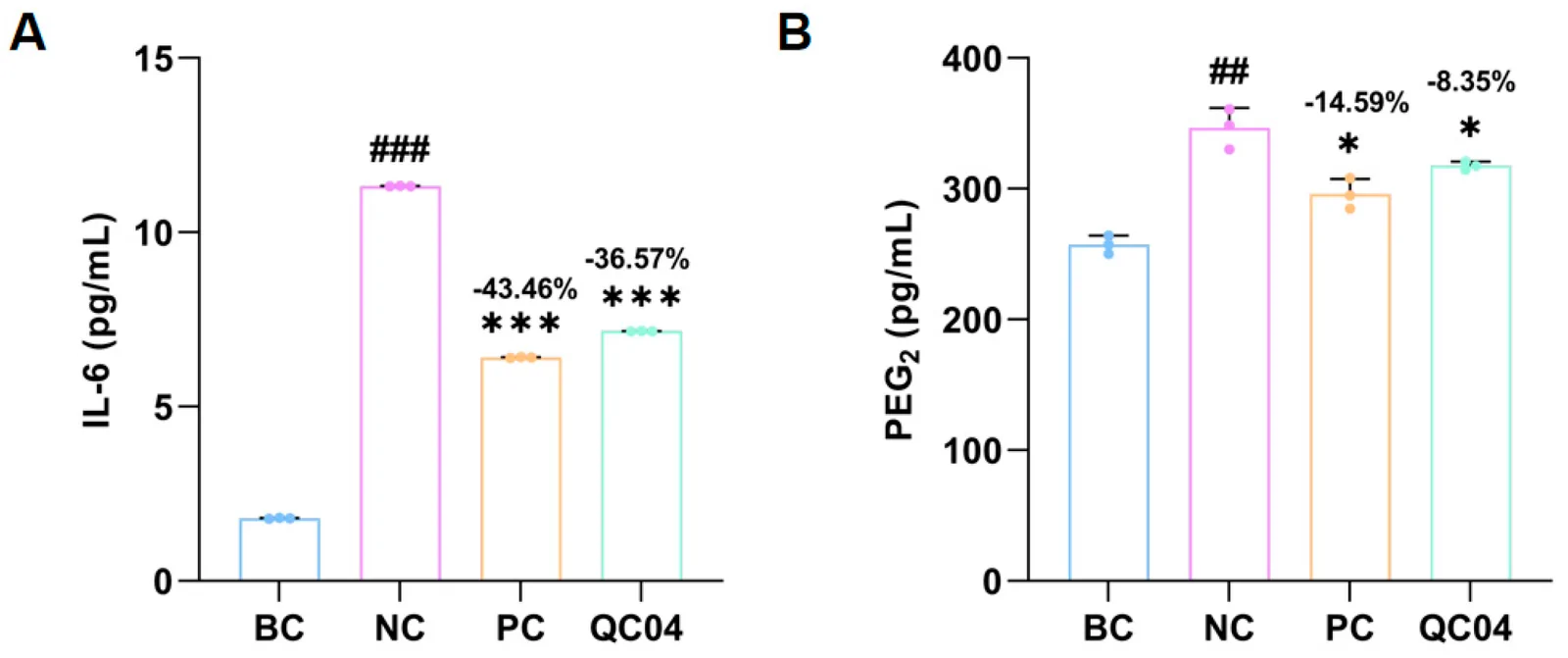
Effects of QC04 on UVB-induced IL-6 and PGE_2_ production in the 3D epidermal model (EpiKutis^®^). (**A**) IL-6 levels in culture supernatants; (**B**) PGE_2_ levels in culture supernatants. BC, blank control; NC, UVB model control; PC, positive control treated with 0.01% dexamethasone; QC04, UVB-exposed model treated with 200 μg/mL QC04. Data are presented as the mean ± SD (*n* = 3 independent tissues per group). ## *p* < 0.01 and ### *p* < 0.001 versus the BC group; * *p* < 0.05 and *** *p* < 0.001 versus the NC group. Each dot represents an independent tissue sample.

All tested concentrations were within the pre-validated safe range, and the 3D epidermal models maintained intact morphology after UVB irradiation and sample treatment. UVB irradiation significantly increased IL-6 secretion in the 3D epidermal model (EpiKutis^®^) compared with the blank control group (*p* < 0.001), confirming the induction of an inflammatory response (Figure 9A). Treatment with QC04 at 200 μg/mL reduced IL-6 secretion by 36.75% relative to the UVB model group (*p* < 0.001), whereas dexamethasone produced an inhibition rate of 43.46% (*p* < 0.001).

UVB exposure also significantly elevated PGE_2_ production (*p* < 0.01; Figure 9B). QC04 reduced PGE_2_ secretion by 8.35% compared with the UVB model group (*p* < 0.05), while dexamethasone decreased PGE_2_ production by 14.59% (*p* < 0.05). These results indicate that QC04 attenuated UVB-induced inflammatory mediator production in the 3D epidermal model.

#### 2.5.3. Effects of QC04 on UVB-Induced TNF-α and NF-κB p65 Expression

**Figure 10:**
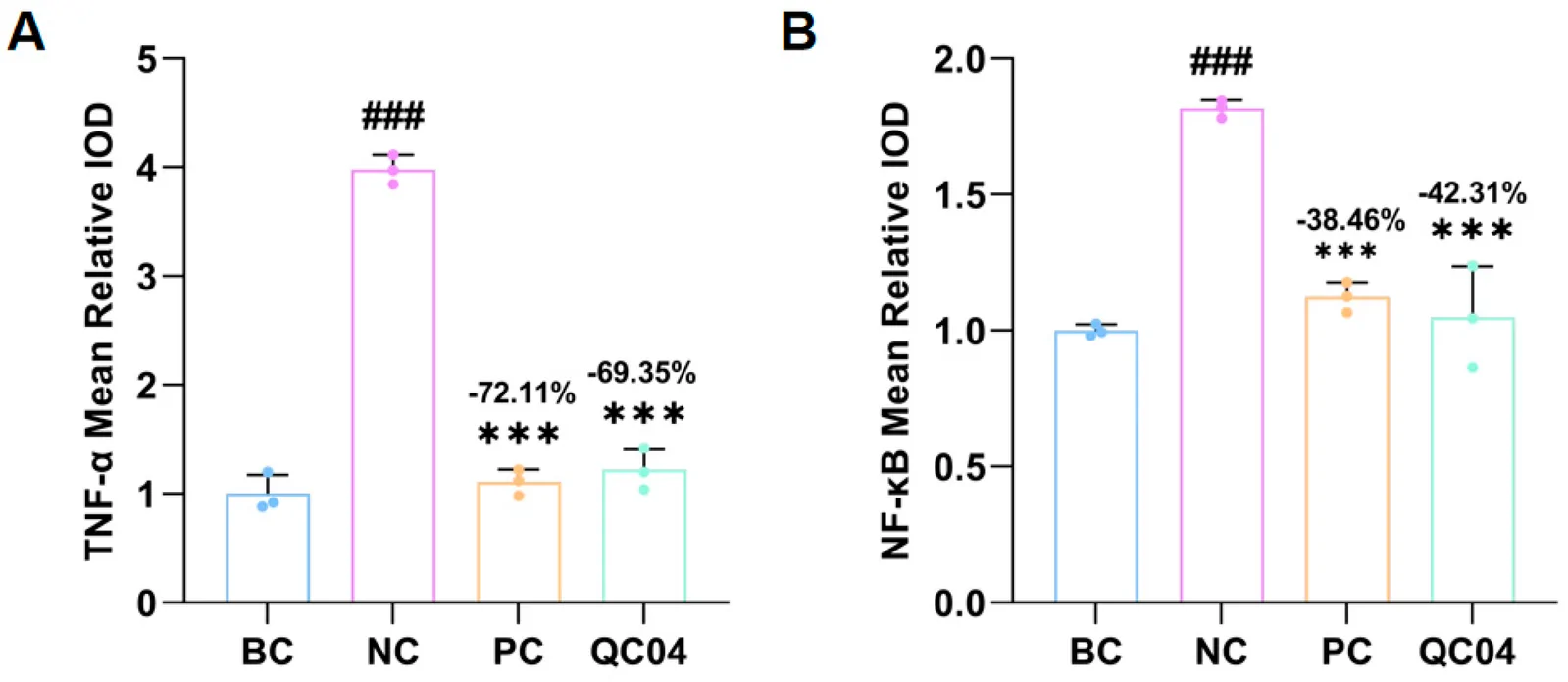

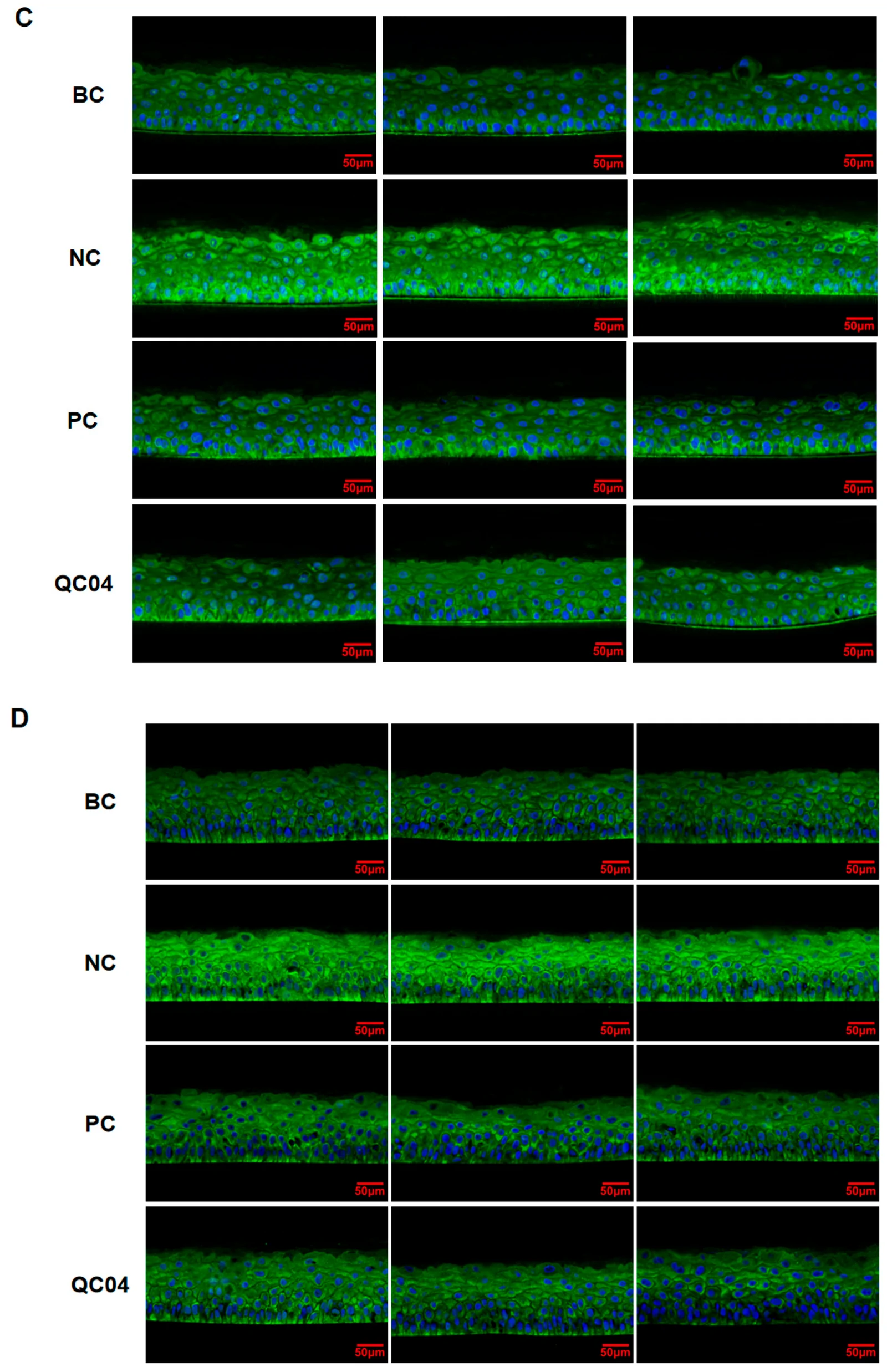
Effects of QC04 on UVB-induced TNF-α and NF-κB p65 expression in the 3D epidermal model (EpiKutis^®^). (**A**) Semi-quantitative analysis of TNF-α fluorescence intensity; (**B**) semi-quantitative analysis of NF-κB p65 fluorescence intensity; (**C**) representative immunofluorescence images of TNF-α; and (**D**) representative immunofluorescence images of NF-κB p65. Target proteins are shown in green, and nuclei are counterstained with DAPI in blue. BC, blank control; NC, UVB model control; PC, positive control treated with 0.01% dexamethasone; QC04, UVB-exposed model treated with 200 μg/mL QC04. Scale bar = 50 μm. Data are presented as the mean ± SD (*n* = 3 independent tissues per group). ### *p* < 0.001 versus the BC group; *** *p* < 0.001 versus the NC group. Each dot represents an independent tissue sample.

Immunofluorescence analysis showed that UVB irradiation markedly increased TNF-α expression in the 3D epidermal model (EpiKutis^®^) compared with the blank control group (*p* < 0.001; Figure 10A,C). Treatment with QC04 at 200 μg/mL reduced TNF-α expression by 69.35% relative to the UVB model group (*p* < 0.001), while dexamethasone produced a 72.11% reduction (*p* < 0.001).

UVB irradiation also significantly increased NF-κB p65 expression (*p* < 0.001; Figure 10B,D). QC04 treatment decreased NF-κB p65 expression by 42.31% compared with the UVB model group (*p* < 0.001), whereas dexamethasone reduced it by 38.46% (*p* < 0.001). These findings indicate that QC04 suppressed UVB-induced TNF-α and NF-κB p65 expression in the 3D epidermal model.

### 2.6. Soothing Effects of QC04 in Zebrafish Models

#### 2.6.1. Effects of QC04 on Copper Sulfate-Induced Neutrophil Recruitment

**Figure 11:**
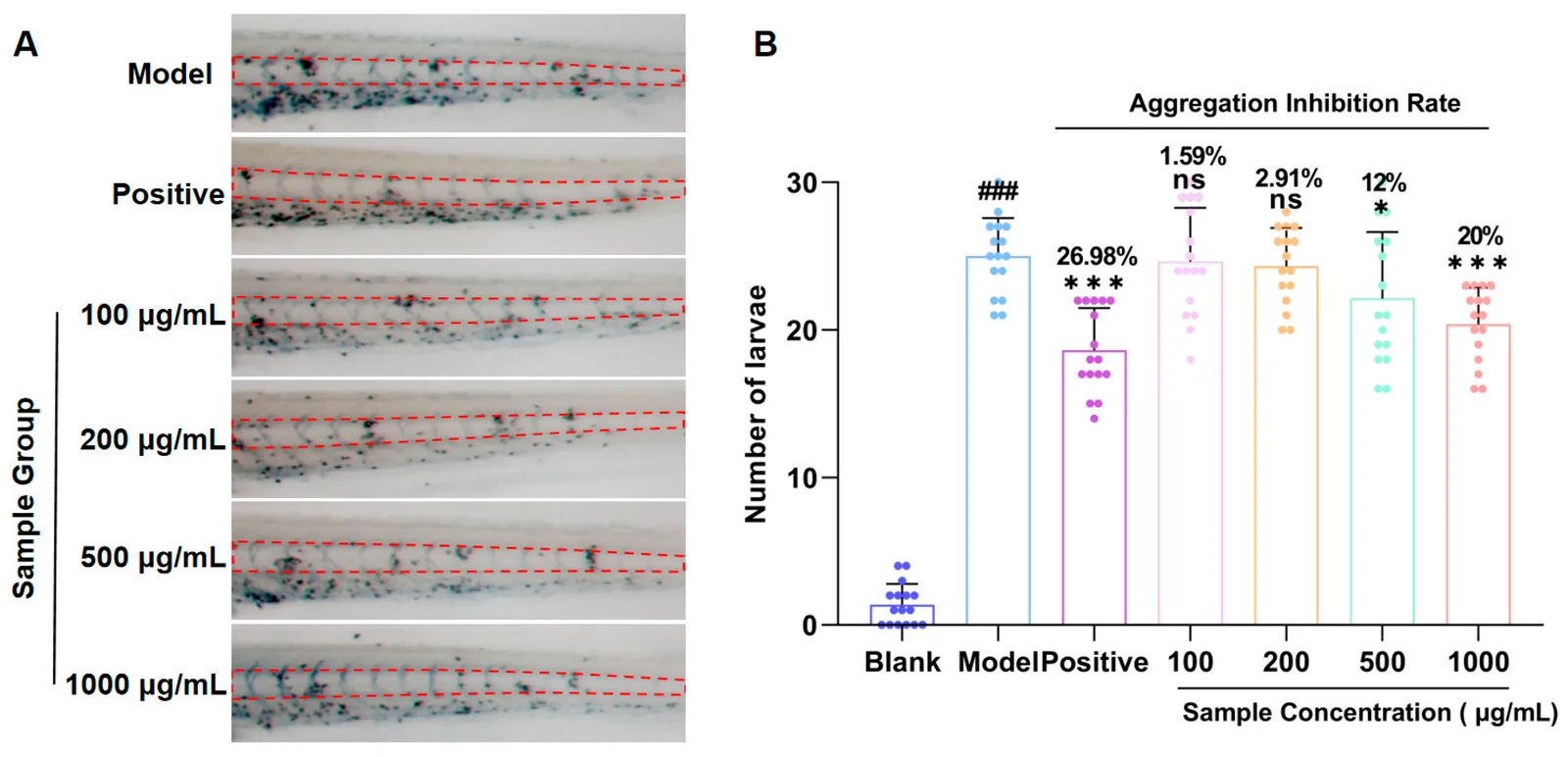
Effects of QC04 on copper sulfate-induced neutrophil recruitment in zebrafish larvae. (**A**) Representative images of Sudan Black B-stained neutrophils in the tail region; red dashed lines indicate the area used for cell counting. (**B**) Quantification of neutrophil numbers and corresponding inhibition rates. Data are presented as the mean ± SD (*n* = 16). ### *p* < 0.001 versus the blank control group; * *p* < 0.05 and *** *p* < 0.001 versus the model control group; ns, not significant. Each dot represents an individual zebrafish larva. The positive group received Indomethacin treatment.

Copper sulfate exposure significantly increased neutrophil recruitment in the tail region of zebrafish larvae compared with the blank control group (*p* < 0.001), confirming successful establishment of the inflammatory model (Figure 11). The mean neutrophil counts were 25.00 ± 2.58 in the model group and 18.63 ± 2.85 in the indomethacin positive-control group. QC04 treatment resulted in mean neutrophil counts of 20.38 ± 2.50, 22.13 ± 4.51, 24.31 ± 2.60, and 24.63 ± 3.65 at 1000, 500, 200, and 100 μg/mL, respectively. At 500 and 1000 μg/mL, QC04 produced inhibition rates of 12.00% (*p* < 0.05) and 20.00% (*p* < 0.001), respectively. In contrast, QC04 at 100 and 200 μg/mL resulted in inhibition rates of 1.59% and 2.91%, with no significant differences from the model group. These results indicate that QC04 exerted a modest inhibitory effect on copper sulfate-induced neutrophil recruitment at 500 and 1000 μg/mL [32].

#### 2.6.2. Effects of QC04 on Histamine-Induced Vasodilation

**Figure 12:**
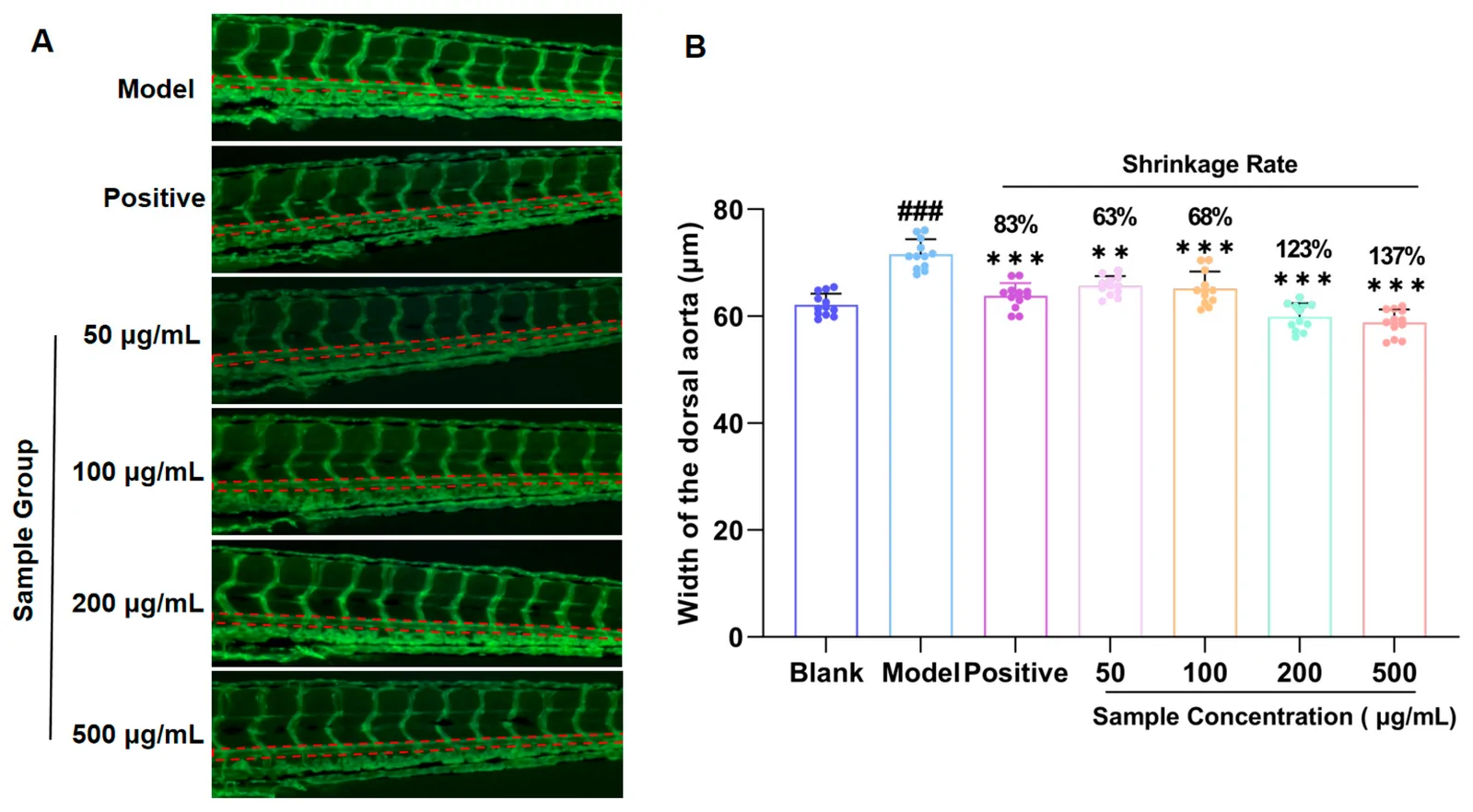
Effects of QC04 on histamine-induced vasodilation in Fli1:EGFP transgenic zebrafish embryos. (**A**) Representative fluorescence images of the dorsal aorta in each treatment group; red dashed lines indicate the region used for vascular width measurement. (**B**) Quantification of dorsal aorta width and the corresponding vascular constriction rates. Data are presented as the mean ± SD (*n* = 12). ### *p* < 0.001 versus the blank control group; ** *p* < 0.01 and *** *p* < 0.001 versus the model control group. Each dot represents an individual zebrafish embryo. The positive group received loratadine treatment.

Histamine exposure significantly increased the width of the dorsal aorta in zebrafish embryos compared with the blank control group (*p* < 0.001), confirming successful establishment of the vasodilation model (Figure 12). QC04 treatment reduced histamine-induced vascular dilation at 500, 200, 100, and 50 μg/mL, with calculated constriction rates of 135%, 123%, 68%, and 63%, respectively. The corresponding mean vessel widths were 58.80 ± 2.34, 59.94 ± 2.41, 65.16 ± 3.05, and 65.67 ± 1.81 μm, respectively, compared with 71.59 ± 2.67 μm in the model group, 62.14 ± 2.01 μm in the untreated control group, and 63.78 ± 2.34 μm in the loratadine positive-control group. The constriction rates above 100% at 500 and 200 μg/mL resulted from the corresponding mean vessel widths being lower than those of the untreated control group. The effects were significant at 50 μg/mL (*p* < 0.01) and at 100–500 μg/mL (*p* < 0.001). These findings provide preliminary evidence that QC04 may attenuate histamine-induced vasodilation in zebrafish embryos [33,34].

## 3. Discussion

Overall, the present study systematically characterized the chemical composition, anti-inflammatory activity, skin-soothing efficacy, and potential molecular mechanisms of QC04, the 75% ethanol fraction derived from *P. utilis* seeds. Importantly, a key feature of this work is the integration of activity-guided fractionation with untargeted metabolomics, network pharmacology, molecular docking, molecular dynamics simulation, a reconstructed human epidermal model, and zebrafish assays. This approach enabled the principal active fraction to be identified from a chemically complex seed extract and linked its metabolite profile to predicted molecular targets and reproducible biological responses. Previous investigations of *P. utilis* have addressed seed oil and its biological activities [7,9], the isolation and characterization of individual phytochemicals [6,10,11,12], antioxidant activity [15], and skin-barrier protection [13,14]. In contrast, the present study focuses on an activity-enriched seed fraction and further connects its chemical profile with predicted molecular targets and functional inflammatory responses at both tissue and whole-organism levels. The present study focused on ethanol-soluble constituents rather than comprehensively characterizing the fixed seed oil. Therefore, its chemical profile may differ from that of the seed oil, particularly in non-polar lipid constituents. Non-polar solvent extraction was not included because comparative solvent extraction was beyond the scope of this study.

Specifically, activity-guided fractionation was employed to identify the fraction most closely associated with the anti-inflammatory activity of the crude extract. Among the four fractions obtained by D101 macroporous resin chromatography, QC04 exhibited the strongest concentration-dependent inhibition of LPS-induced NO production, with an IC_50_ value of 239.21 ± 6.70 μg/mL. Although cytotoxicity was observed at the highest concentration tested, QC04 retained clear NO-inhibitory activity within the non-cytotoxic concentration range and showed substantially greater potency than the other fractions. Collectively, these findings indicate that the 75% ethanol eluate effectively enriched constituents associated with anti-inflammatory activity and provide a rational basis for selecting QC04 for subsequent chemical characterization and mechanistic investigation. This finding is broadly consistent with previous evidence for the anti-inflammatory activity of *P. utilis* seed-derived preparations. For example, Chauhan et al. reported an IC_50_ of 518.14 μg/mL for *P. utilis* seed oil in a bovine serum albumin denaturation assay and observed significant anti-inflammatory effects at 200 mg/kg in rat models [7]. Although these values cannot be directly compared with the present IC_50_ because of differences in extraction chemistry, biological models, and activity endpoints, both studies support the anti-inflammatory potential of seed-derived *P. utilis*. Notably, the present activity-guided fractionation further narrows the active chemical fraction of the seeds and provides a basis for subsequent mechanistic investigation.

Untargeted metabolomic profiling identified 1039 metabolites in QC04, with phenylpropanoids and lipids representing the predominant chemical classes. This finding is in agreement with previous phytochemical investigations showing that *P. utilis* contains diverse flavonoids, phenolic acids, triterpenoids, and other specialized metabolites in different plant tissues [6,9,10,11,12,15]. The representative metabolites selected for further analysis included flavonoids, phenolic acids, procyanidins, triterpenoids, and saponins [9,19]. Notably, several constituents detected in QC04, including epigallocatechin gallate, kaempferol, procyanidins, euscaphic acid, polygalacic acid, and madecassic acid, have been associated with the regulation of inflammatory mediator production, oxidative stress, and UV-related skin injury [16,35]. The detection of these structurally diverse metabolites is generally consistent with previous phytochemical studies of *P. utilis*, which have reported flavonoids, triterpenoids, phenolic compounds, and other secondary metabolites in its seeds and other tissues [6,9,10,11,12,16]. However, the present study characterizes these constituents within an activity-enriched fraction rather than focusing on isolated compounds or seed oil alone. The coexistence of these structurally diverse metabolites provides a plausible chemical basis for the broad biological activity of QC04 and is consistent with the multicomponent and multi-target characteristics of plant-derived preparations. Rather than being attributable to a single constituent, the activity of QC04 is likely derived from the coordinated contribution of multiple bioactive metabolites.

Network pharmacology identified AKT1, MAPK1, MAPK8, and MAPK14 as core targets of QC04, with TNF and MAPK signaling among the major enriched pathways. These kinases can regulate NF-κB-dependent inflammatory responses, while TNF-α further amplifies MAPK and NF-κB signaling, ultimately promoting the expression of TNF, IL6, and PTGS2 and increasing TNF-α, IL-6, and PGE_2_ production [26,27]. This predicted relationship among AKT1/MAPK signaling, TNF-α/NF-κB regulation, and downstream inflammatory mediators provided the rationale for selecting TNF-α, NF-κB p65, IL-6, and PGE_2_ as experimental readouts. Molecular docking showed favorable interactions between representative QC04 metabolites and AKT1, MAPK1, MAPK8, and MAPK14, with binding energies ranging from −10.2 to −8.6 kcal/mol. The 100 ns molecular dynamics simulation further demonstrated the conformational stability of the euscaphic acid–AKT1 complex [36]. Together, these computational findings provide structural plausibility for the predicted target interactions and generate a testable mechanistic hypothesis that requires further experimental validation. Moreover, the subsequent biological experiments assessed downstream inflammatory markers rather than AKT/MAPK phosphorylation, activity, or direct target engagement. Therefore, these findings should be interpreted as supportive evidence for the predicted involvement of AKT/MAPK-associated pathways rather than direct mechanistic validation.

To assess the biological relevance of these predictions, the UVB-induced 3D epidermal model (EpiKutis^®^) was used. At the non-cytotoxic concentration of 200 μg/mL, QC04 markedly reduced TNF-α and NF-κB p65 expression and simultaneously decreased IL-6 and PGE_2_ secretion. These markers represent interconnected components of the predicted inflammatory network, in which AKT1 and MAPKs have been implicated as upstream kinase regulators, while TNF-α, NF-κB, IL-6, and PGE_2_ represent inflammatory mediators at downstream levels [27,28]. The coordinated reduction in TNF-α, NF-κB p65, IL-6, and PGE_2_ provides experimental support for the inflammatory network predicted by the computational analyses and is consistent with a role for the AKT/MAPK–TNF-α/NF-κB-associated axis in the anti-inflammatory activity of QC04. The use of a reconstructed human epidermal model also strengthens the physiological relevance of the findings because it retains the stratified architecture and tissue-level responsiveness of human epidermis more effectively than conventional monolayer cultures [20]. Notably, QC04 reduced TNF-α and NF-κB p65 expression under the present experimental conditions, supporting its anti-inflammatory activity at the tissue level. Previous studies have also reported skin-related effects of *P. utilis* seed-derived preparations in reconstructed skin models, including improvement of skin-barrier properties by seed oil and promotion of epidermal barrier repair by oil-cake extracts [13,14]. In contrast, the present study focused on UVB-induced inflammatory responses and demonstrated coordinated suppression of TNF-α, NF-κB p65, IL-6, and PGE_2_, suggesting a distinct anti-inflammatory profile of QC04.

The zebrafish assays extended the biological evaluation of QC04 to the whole-organism level. QC04 inhibited copper sulfate-induced neutrophil recruitment at 500 and 1000 μg/mL, indicating suppression of acute inflammatory cell migration. It also reduced histamine-induced vasodilation across all tested concentrations. The calculated constriction rates exceeded 100% at 500 and 200 μg/mL because the corresponding mean vessel widths were lower than those of the untreated control group; these values reflect the relative calculation based on the model-to-control range. Therefore, the vascular responses at higher concentrations should be interpreted cautiously, as nonspecific vasoconstrictive effects, altered circulation, or concentration-dependent toxicity cannot be completely excluded. These two models reflect distinct components of acute inflammation: neutrophil recruitment represents inflammatory cell activation and migration [32,37], whereas histamine-induced vasodilation reflects rapid vascular responses associated with redness and irritation [8,9]. This complements previous studies of *P. utilis* seed oil, in which anti-inflammatory activity was demonstrated using protein-denaturation and rodent paw-edema models [7], but provides additional evidence based on direct evaluation of inflammatory cell recruitment and vascular responses in vivo. Notably, the consistent effects observed in both zebrafish models support the biological activity of QC04, which is not limited to changes in individual inflammatory mediators but extends to functional inflammatory responses in vivo. Together with the findings from the reconstructed human epidermal model, these results provide complementary evidence supporting the potential anti-inflammatory and soothing effects of QC04 at both the tissue and whole-organism levels.

From an ethnopharmacological perspective, the traditional topical use of *P. utilis* seed-derived preparations provides an important context for investigating the biological potential of seed-derived constituents. More importantly, the study provides a coherent research framework linking active-fraction screening, chemical profiling, target prediction, molecular interaction analysis, reconstructed human epidermal validation, and whole-organism functional assessment. This multilevel evidence strengthens the biological interpretation of QC04 and highlights its potential biological activity relevant to skin-soothing and post-UV inflammatory responses. Accordingly, these findings contribute both to the scientific interpretation of traditional knowledge and to the value-added utilization of *P. utilis* seed resources.

Despite these promising findings, several limitations should be acknowledged. Comprehensive metabolomic profiling was performed only for QC04, the fraction showing the strongest anti-inflammatory activity; therefore, the present study cannot directly compare the chemical compositions of QC01–QC04 or definitively identify metabolites specifically enriched in QC04. Although the preparation yield of QC04 was determined, batch-to-batch reproducibility, quantitative marker determination, and a validated chemical fingerprint were not established in the present study. Moreover, QC04 remains a multicomponent fraction, and the individual contributions of its metabolites to the observed effects have not been fully determined. The relative LC-MS peak areas provide semi-quantitative information within the present dataset and should not be interpreted as directly comparable absolute abundances across different compounds because of compound-specific ionization responses. The selection of representative metabolites also considered previously reported biological activities as contextual information, which may introduce selection bias; therefore, the network pharmacology and molecular docking results should be interpreted as hypothesis-generating evidence rather than direct mechanistic validation. The molecular docking and molecular dynamics results provide supportive evidence, but direct target engagement and regulation of AKT/MAPK signaling require further experimental validation, such as phosphorylation analysis and target-specific intervention. In addition, only the highest non-cytotoxic concentration was tested in the 3D epidermal model. A dedicated quantitative assessment of tissue viability and integrity after UVB exposure and treatment was not conducted. Moreover, NF-κB p65 was only semi-quantified via total-protein immunofluorescence without assessment of phosphorylation or nuclear translocation, which cannot directly reflect pathway activation status. The present study also did not evaluate skin penetration, epidermal-barrier outcomes, mammalian or human skin efficacy, or extended safety; therefore, the potential of QC04 for post-UV repair skincare remains to be further investigated. Multi-concentration dose–response and targeted mechanistic experiments are therefore warranted to improve conclusion robustness. In the zebrafish assays, only viable and morphologically normal embryos meeting the ≥90% survival criterion were included in the final analysis. However, morphology and circulation were not separately quantified, and independent clutch-level replication was not performed; thus, nonspecific vascular effects or concentration-dependent toxicity at higher QC04 concentrations cannot be completely excluded. Future studies should therefore prioritize comparative chemical characterization and batch reproducibility across fractions, isolation and characterization of key active constituents, multi-concentration validation in the 3D epidermal model, and further mechanistic validation in mammalian skin models.

## 4. Materials and Methods

### 4.1. Materials and Reagents

The seeds of *P. utilis* Royle were collected from Zhanhe Township, Ninglang County, Lijiang, Yunnan Province, China. The plant material was authenticated by Dr. Lixin Yang at the Herbarium of the Kunming Institute of Botany, Chinese Academy of Sciences, where a voucher specimen (No. 1644011) was deposited.

D101 macroporous resin and phosphate-buffered saline (PBS) were purchased from Solarbio (Beijing, China). RAW 264.7 macrophages were obtained from the Cell Bank of the Chinese Academy of Sciences (Shanghai, China). Dulbecco’s modified Eagle’s medium (DMEM) and fetal bovine serum (FBS) were supplied by VivaCell Biosciences (Shanghai, China). Griess reagent, lipopolysaccharide (LPS, Escherichia coli O111), L-NMMA, MTT, and dimethyl sulfoxide (DMSO) were purchased from Sigma-Aldrich (St. Louis, MO, USA).

Human keratinocytes (Lot No. Ep26012804), 3D epidermal model (EpiKutis^®^) (Lot No. ES260605), KcGrowth and EpiGrowth medium were provided by Guangdong Bosi Biotechnology Co., Ltd. Shaanxi Branch (Xi’an, China). Dexamethasone was obtained from the National Institutes for Food and Drug Control (Beijing, China). The IL-6 enzyme-linked immunosorbent assay (ELISA) kit was purchased from ABclonal (Wuhan, China), and the PGE_2_ ELISA kit was obtained from Enzo Life Sciences (Farmingdale, NY, USA). Anti-TNF-α and anti-NF-κB p65 primary antibodies were purchased from Abcam (Cambridge, MA, USA). DAPI and Alexa Fluor 488-conjugated goat anti-rabbit IgG secondary antibodies were obtained from Thermo Fisher Scientific (Waltham, MA, USA), while paraformaldehyde was purchased from Biosharp (Hefei, China).

Copper sulfate, histamine, indomethacin, loratadine, Sudan Black B, and other analytical-grade reagents were purchased from Sinopharm Chemical Reagent Co., Ltd. (Shanghai, China). Wild-type AB strain and Fli1 transgenic zebrafish embryos were obtained from the Zebrafish International Resource Center (ZIRC), University of Oregon, Eugene, OR, USA.

### 4.2. Instruments

The ultra-high-performance liquid chromatography system (Vanquish UHPLC), Orbitrap Exploris 120 high-resolution mass spectrometer, Heraeus Fresco 17 refrigerated centrifuge, and 150I CO_2_ incubator were purchased from Thermo Fisher Scientific (Waltham, MA, USA). The analytical balance (BSA124S-CW) was obtained from Sartorius (Göttingen, Germany). An ultrasonic extractor (PS-60AL), tissue homogenizer (JXFSTPRP-24), and freeze dryer (LGJ-10C) were purchased from Shenzhen Leidebang Electronics Co., Ltd., Shanghai Jingxin Technology Co., Ltd., and Sihuan Furui Keyi Technology Development Co., Ltd., respectively. A laminar flow cabinet (SW-CJ-2F) was obtained from Sujing Antai (Suzhou, China). A microplate reader (Epoch), a UVB irradiation system, an inverted microscope (CKX53), and a fluorescence microscope (BX43) were obtained from BioTek (Winooski, VT, USA), Philips (Amsterdam, The Netherlands), and Olympus (Tokyo, Japan), respectively.

### 4.3. Preparation of P. utilis Seed Extract Fractions

#### 4.3.1. Preparation of the Crude Ethanol Extract

Dried *P. utilis* seeds (3.0 kg) were pulverized and extracted three times with 95% ethanol using ultrasound-assisted extraction at a solid-to-liquid ratio of 1:5 (*w*/*v*), an ultrasonic power of 300 W, and a temperature of 50 °C for 60 min per extraction. The combined extracts were filtered and concentrated under reduced pressure at 50 °C to obtain the crude ethanol extract. The crude extract yield was calculated based on the initial dry weight of the seeds. The seeds were extracted with ethanol to obtain a broad range of polar and moderately polar constituents from the seed material. Ethanol was selected because it is suitable for recovering diverse secondary metabolites and is compatible with the subsequent D101 macroporous resin fractionation.

#### 4.3.2. Fractionation Using D101 Macroporous Resin

The crude ethanol extract was dissolved in methanol and loaded onto a pretreated and equilibrated D101 macroporous resin column. After static adsorption at room temperature for 2 h, the column was sequentially eluted with deionized water and 25%, 50%, and 75% aqueous ethanol at a flow rate of 2 bed volumes per hour (BV/h). The corresponding eluates were collected separately and concentrated to dryness under reduced pressure at 50 °C to obtain the water fraction (QC01), 25% ethanol fraction (QC02), 50% ethanol fraction (QC03), and 75% ethanol fraction (QC04), and the yield of each fraction was calculated based on the mass of the loaded crude extract. All fractions were stored in airtight containers at low temperature until further analysis.

### 4.4. Activity-Guided Screening of the Anti-Inflammatory Fraction

The inhibitory effects of QC01-QC04 on nitric oxide (NO) production were evaluated in lipopolysaccharide (LPS)-stimulated RAW 264.7 macrophages. All samples were dissolved in dimethyl sulfoxide (DMSO), with a final DMSO concentration of <0.1% (*v*/*v*) across all groups to eliminate solvent interference. Cells were seeded in 96-well plates and simultaneously treated with LPS (1 μg/mL) and each fraction at concentrations of 50, 100, 200, 400, and 800 μg/mL. Cells treated with LPS alone served as the model control, and L-NMMA was used as the positive control. After overnight incubation, nitrite accumulation in the culture supernatants was quantified using the Griess assay, and absorbance was measured at 570 nm. Cell viability was evaluated in parallel using the MTS assay to exclude cytotoxic interference [38]. The inhibition rate of NO production was calculated according to Equation (1):

(1) $$\mathrm{NO}\;\mathrm{inhibition}\;\mathrm{rate}\;(\%)=\frac{{\mathrm{OD}}_{\mathrm{model}}-{\mathrm{OD}}_{\mathrm{sample}}}{{\mathrm{OD}}_{\mathrm{model}}}\times 100\%$$

where OD_model_ and OD_sample_ denote the absorbance values of the LPS-treated model control and sample-treated groups, respectively.

IC_50_ values were calculated via the two-point logarithmic interpolation method, using the two adjacent concentrations spanning 50% inhibition under the 2-fold dilution gradient. Cell viability was measured in parallel with NO measurement. Concentrations with cell viability ≥90% were defined as non-cytotoxic, and only inhibitory effects within this range were interpreted as specific anti-inflammatory activity. This experiment was conducted with one biological replicate and three technical replicates.

### 4.5. Untargeted Metabolomics Analysis

QC04 (approximately 10 mg) was extracted with 1 mL of pre-cooled methanol-acetonitrile-water (2:2:1, *v*/*v*/*v*) containing internal standards. Samples were homogenized at 35 Hz for 4 min and sonicated in an ice-water bath for 5 min, repeated three times. After incubation at −40 °C for 1 h, samples were centrifuged at 13,800× *g* for 15 min at 4 °C, and the supernatants were collected for analysis. Metabolomic profiling was performed using a Vanquish UHPLC system coupled to an Orbitrap Exploris 120 mass spectrometer. Polar and non-polar metabolites were separated using an ACQUITY UPLC BEH Amide column and a Kinetex C18 column, respectively. Mass spectra were acquired in positive- and negative-ionization modes at full-scan and MS/MS resolutions of 60,000 and 15,000, respectively, with stepped collision energies of 20, 30, and 40. Raw data were converted to mzXML format using ProteoWizard 3.0.24054 and processed using an in-house R-based workflow. Metabolites were annotated against BiotreeDB 4.0 and the plant-specific BT-Plant 1.1 database. BiotreeDB 4.0, the primary database used for annotation, is an in-house spectral library generated from authentic chemical reference standards analyzed under identical chromatographic and mass spectrometric conditions to those applied to the experimental samples. Metabolite identification confidence was classified strictly following the minimum reporting standards of the Metabolomics Standards Initiative (MSI) [22]. Metabolites assigned MSI Level 1 confidence were confirmed via three orthogonal analytical dimensions: accurate precursor mass, chromatographic retention time, and MS/MS fragmentation profile. The predefined acceptance criteria for Level 1 identification included a precursor mass error ≤2 ppm, a retention time deviation ≤0.3 min, and an MS/MS spectral matching score ≥3.95. Relative peak area values reported in this study correspond to TIC-normalized ion-count intensities. Values displayed in Table 1 are scaled by a factor of 10^−6^ for readability, as marked by the unit label (×10^6^) [39].

### 4.6. Network Pharmacology, Molecular Docking, and Molecular Dynamics Simulation

Potential targets of the representative metabolites were predicted using the PharmMapper v2 server (accessed 22 May 2026; Norm Fit score > 0.7; *Homo sapiens*), and inflammation-related targets were retrieved from GeneCards (accessed 22 May 2026; Relevance Score cutoff applied). All identifiers were mapped to UniProt IDs (*Homo sapiens*) and deduplicated before set intersection. Overlapping targets were submitted to STRING (confidence > 0.7; full human proteome as statistical background; disconnected nodes removed), and the network was analyzed in Cytoscape 3.10.4. Hub targets were defined as genes ranking in the top 10% by Degree, Betweenness Centrality and Closeness Centrality (CytoHubba plugin). GO and KEGG enrichment analyses were performed in DAVID (Homo sapiens background; Benjamini–Hochberg-adjusted *p* < 0.05), with results visualized in R.

Six representative metabolites and four core targets (PDB IDs: 3QKK, 5NZZ, 3V3V, 8PT1) were selected for docking. Ligand structures were from PubChem; protein crystal structures were from the Protein Data Bank. Proteins and ligands were prepared in OpenBabel v3.2.1 (cofactors/crystallographic waters removed; protonation assigned at pH 7.4), with full grid box parameters listed in Supplementary Table S3. Docking was run in AutoDock Vina 1.2.5 (exhaustiveness = 32; 3 independent runs per ligand–protein pair), and the lowest-energy pose was selected for analysis. Native ligand re-docking validation (2.0 Å RMSD acceptability threshold) was performed, with corresponding limitations noted in the manuscript. Optimal conformations were visualized in PyMOL 2.6.0a0 (open-source build) and analyzed via PLIP [30].

Molecular dynamics simulations of the euscaphic acid–AKT1 complex were performed in GROMACS 2020.06. The ligand was parameterized with GAFF atom types via Sobtop v1.0; the protein used the Amber ff14SB force field and water used the TIP3P model [31]. The system was solvated in a cubic periodic box (~8.86 nm edge; charge-neutralized with Na^+^/Cl^−^), energy-minimized, and equilibrated via sequential 2 ns NVT and 2 ns NPT stages with heavy-atom position restraints. Two independent 100 ns NPT production runs (2 fs timestep; V-rescale thermostat; Berendsen barostat; PME for long-range electrostatics) were performed with distinct random velocity seeds. All analyses (RMSD, RMSF, radius of gyration, SASA, hydrogen bonds, free-energy landscape via g_sham) were conducted on the 25–100 ns equilibrated trajectory segment.

### 4.7. Anti-Inflammatory Activity in the 3D Epidermal Model (EpiKutis^®^)

#### 4.7.1. Cytotoxicity Assay

The cytotoxicity of QC04 toward human keratinocytes was evaluated using the MTT assay [36]. QC04 was dissolved in dimethyl sulfoxide (DMSO), with a final DMSO concentration of <0.1% (*v*/*v*) across all groups. Cells were treated with QC04 at 12.5–1000 μg/mL for 24 h, followed by incubation with MTT (0.5 mg/mL) for 4 h. Culture medium with an equal volume of DMSO served as the vehicle control. The resulting formazan crystals were dissolved in DMSO, and absorbance was measured at 490 nm. Cell viability was calculated as follows:

(2) $$Cellviability(\%)=\frac{{OD}_{sample}-{OD}_{blank}}{{OD}_{vehicle}-{OD}_{blank}}\times 100\%$$

where OD_sample_, OD_vehicle_, and OD_blank_ represent the absorbance values of the QC04-treated group, vehicle control group, and blank well, respectively.

#### 4.7.2. UVB-Induced Inflammatory Response

3D epidermal models (EpiKutis^®^) were divided into blank control, UVB model, positive control, and QC04 groups, with three independent tissues per group. Each tissue was treated as an independent experimental unit. QC04 was dissolved in DMSO and diluted with EpiGrowth medium, with a final DMSO concentration <0.1% (*v*/*v*). The UVB model group received medium with an equal volume of DMSO as the vehicle control. Except for the blank control, all models were exposed to UVB irradiation at 600 mJ/cm^2^. QC04 was administered immediately before UVB irradiation, representing a preventive (pre-treatment) exposure protocol. The positive control and QC04 groups were treated with 0.01% dexamethasone and 200 μg/mL QC04, respectively. After 24 h, culture supernatants were collected, and IL-6 and PGE_2_ levels were measured using commercial ELISA kits [20,40].

#### 4.7.3. Immunofluorescence Analysis

After treatment, the epidermal tissues were fixed with 4% paraformaldehyde, permeabilized, blocked, and incubated overnight at 4 °C with primary antibodies against TNF-α and NF-κB p65. The tissues were then incubated with an Alexa Fluor 488-conjugated secondary antibody and counterstained with DAPI. Images were acquired using a fluorescence microscope (Olympus BX43, 200×), and fluorescence intensity was quantified using Fiji-ImageJ, version 1.54p [37]. Three independent tissue samples were analyzed per group. Measurements obtained from images within the same tissue were summarized at the tissue level and were not treated as independent observations. Image acquisition and quantitative analysis were performed in a blinded manner.

### 4.8. Evaluation of Soothing Activity in Zebrafish Models

#### 4.8.1. Copper Sulfate-Induced Neutrophil Recruitment Assay

Wild-type AB zebrafish larvae at 3 days post-fertilization (dpf) were randomly assigned to six groups. Twenty-four larvae were initially assigned to each group to compensate for potential mortality and malformation. QC04 was dissolved in DMSO, with a final DMSO concentration ≤ 0.1% (*v*/*v*) in embryo medium. The copper sulfate model group received an equal volume of DMSO as the vehicle control. Larvae were exposed to copper sulfate (10 μM) alone or together with QC04 at 100, 200, 500, or 1000 μg/mL for 40 min. This represents a co-treatment exposure protocol, with QC04 administered simultaneously with the stimulus. Larvae maintained in embryo culture medium served as the blank control, while those treated with copper sulfate and indomethacin (10 μM) served as the positive control. Following exposure, the larvae were fixed and stained with Sudan Black B. Neutrophils in the tail lateral-line region were counted under a microscope, and the inhibition of neutrophil recruitment was calculated relative to the copper sulfate-treated model group [33,41]. Only larvae with a survival rate of 90% or higher, normal morphology, and acceptable image quality were retained, and 16 larvae were randomly selected from each group for the final statistical analysis (*n* = 16).

#### 4.8.2. Histamine-Induced Vasodilation Assay

The effect of QC04 on histamine-induced vasodilation was evaluated using Fli1:EGFP transgenic zebrafish embryos [34,42]. Embryos at 1 dpf were exposed to histamine (20 μM) for 48 h and subsequently treated with QC04 at 50, 100, 200, or 500 μg/mL for 2 h at 28 °C. QC04 was dissolved in DMSO, with a final DMSO concentration ≤ 0.1% (*v*/*v*). The histamine model group received an equal volume of DMSO as the vehicle control. This represents a therapeutic (post-treatment) exposure protocol, with QC04 administered after vasodilation was established. Fourteen embryos were initially assigned to each group to compensate for potential mortality and malformation. Embryos without histamine exposure served as the blank control, those exposed to histamine alone served as the model control, and loratadine (20 μM) was used as the positive control. After treatment, the embryos were anesthetized and imaged under a fluorescence microscope. The width of the dorsal aorta was measured at five standardized sites per embryo using ImageJ, and the five measurements were averaged to obtain one embryo-level value for statistical analysis. Only larvae with a survival rate of 90% or higher, normal morphology, and acceptable image quality were retained, and 12 embryos were randomly selected from each group for the final statistical analysis (*n* = 12). The vascular constriction rate was calculated relative to the histamine-treated model group. The vascular constriction rate was calculated as follows:

(3) $$\mathrm{Constriction}\;\mathrm{rate}\;(\%)=\frac{M-S}{M-B}\times 100\%\;$$

where M represents the mean vessel width of the model control group, S represents the mean vessel width of the sample-treated group, and B represents the mean vessel width of the untreated control group.

### 4.9. Data Statistics and Analysis

Statistical analyses were performed using IBM SPSS Statistics 27.0.1. Two-tailed independent-samples t-tests were used for between-group comparisons. Data are presented as mean ± SD. A two-sided *p* < 0.05 was considered statistically significant. Figures were prepared using GraphPad Prism 9.5.0.

## 5. Conclusions

Initially, activity-guided fractionation identified QC04, the 75% ethanol eluate of *P. utilis* seed extract, as the fraction with the strongest inhibitory effect on LPS-induced nitric oxide production. Subsequently, untargeted metabolomic profiling revealed that QC04 contains a chemically diverse mixture of bioactive constituents, particularly phenylpropanoids, lipids, and terpenoids. Representative flavonoids, phenolic acids, procyanidins, triterpenoids, and saponins were further selected for mechanistic analysis. Furthermore, integrated network pharmacology, molecular docking, and molecular dynamics simulation implicated an inflammatory regulatory network centered on AKT1, MAPK1, MAPK8, and MAPK14 and supported the potential interactions of representative QC04 metabolites with these targets. These computational findings supported the predicted involvement of these targets and pathways and provided a basis for subsequent biological assessment. To assess the biological relevance of these in silico predictions, the biological relevance of these predictions was further demonstrated in a UVB-induced 3D epidermal model (EpiKutis^®^), in which QC04 downregulated TNF-α and NF-κB p65 expression and reduced the secretion of IL-6 and PGE_2_. In addition, in vivo zebrafish assays showed that QC04 modestly suppressed neutrophil recruitment and attenuated histamine-induced vasodilation. Together, these tissue-level and whole-organism findings provide complementary experimental support for the predicted inflammatory network and strengthen the pharmacological interpretation of QC04 activity. Overall, this study provides a coherent multilevel evidence chain linking activity-guided screening, chemical composition, predicted molecular targets, and reproducible anti-inflammatory and skin-soothing effects. These findings provide experimental evidence for the biological potential of *P. utilis* seed-derived QC04 and support its further preclinical investigation in relation to skin-soothing and post-UV inflammatory responses.

## Figures and Tables

**Table 1:** Twenty representative metabolites selected from QC04 for subsequent network pharmacology analysis.

Compound Name	Score	Level	mz	rt (s)	Formula	Super Class	Class	Relative Peak Area (×10^6^)
Calenduloside E	3.99	Level 1	631.3848	137.2	C_36_H_56_O_9_	Terpenoids	Saponins	17.30 ± 0.34
Urolithin M6	3.99	Level 1	259.025	201.4	C_13_H_8_O_6_	Phenylpropanoids	Coumarins and derivatives	267.66 ± 102.97
Polygalacic acid	3.98	Level 1	503.3377	47.9	C_30_H_48_O_6_	Terpenoids	Triterpenoids	272.03 ± 4.76
Epigallocatechin Gallate	3.98	Level 1	457.078	157.1	C_22_H_18_O_11_	Phenylpropanoids	Flavonoids	5.66 ± 0.06
Kaempferol	3.98	Level 1	285.0401	23.8	C_15_H_10_O_6_	Phenylpropanoids	Flavonoids	882.59 ± 32.97
Euscaphic acid	3.97	Level 1	487.3426	23.2	C_30_H_48_O_5_	Terpenoids	Triterpenoids	179.28 ± 7.63
4-Hydroxybenzoic acid	3.97	Level 1	137.0242	95.9	C_7_H_6_O_3_	Phenylpropanoids	Phenolic acids	92.02 ± 0.21
Pyrocatechol	3.97	Level 1	109.0296	56.5	C_6_H_6_O_2_	Phenylpropanoids	Other Phenylpropanoids	19.88 ± 0.27
Procyanidin B4	3.96	Level 1	577.1346	132.6	C_30_H_26_O_12_	Phenylpropanoids	Anthocyanidins	303.91 ± 6.50
Platycodigenin	3.96	Level 1	519.3326	97.3	C_30_H_48_O_7_	Terpenoids	Triterpenoids	58.64 ± 5.04
Madecassic acid	3.96	Level 1	503.3387	251	C_30_H_48_O_6_	Terpenoids	Triterpenoids	57.45 ± 2.65
Isoquercitrin	3.96	Level 1	465.1023	180.5	C_21_H_20_O_12_	Phenylpropanoids	Flavonoids	146.85 ± 4.47
Nothofagin	3.96	Level 1	435.1298	182.7	C_21_H_24_O_10_	Phenylpropanoids	Flavonoids	135.22 ± 2.21
Epicatechin	3.96	Level 1	291.0861	139	C_15_H_14_O_6_	Phenylpropanoids	Flavonoids	50.59 ± 1.13
Sulfuretin	3.96	Level 1	269.0453	23.2	C_15_H_10_O_5_	Phenylpropanoids	Flavonoids	89.12 ± 2.21
Narcissin	3.95	Level 1	623.1615	146.9	C_28_H_32_O_16_	Phenylpropanoids	Flavonoids	226.92 ± 2.25
Lonicerin	3.95	Level 1	595.1655	149.2	C_27_H_30_O_15_	Phenylpropanoids	Flavonoids	19.13 ± 0.42
Procyanidin B2	3.95	Level 1	577.1349	112.3	C_30_H_26_O_12_	Phenylpropanoids	Anthocyanidins	24.97 ± 2.01
Medicagenic acid	3.95	Level 1	501.323	273	C_30_H_46_O_6_	Terpenoids	Triterpenoids	105.01 ± 4.61
Cacticin	3.95	Level 1	479.1183	185.7	C_22_H_22_O_12_	Phenylpropanoids	Flavonoids	43.34 ± 0.79

Note: Relative peak areas are semi-quantitative liquid chromatography–mass spectrometry (LC-MS) signals from this dataset that have been normalized using total ion current (TIC) profiles (scaled by a factor of 10^6^); they are not intended for direct comparison of the absolute abundances of different compounds.

## Data Availability

The original contributions presented in this study are included in the article/Supplementary Materials. Further inquiries can be directed to the corresponding author.

## Supplementary Materials

The following supporting information can be downloaded at https://www.mdpi.com/article/10.3390/ijms27177951/s1.

## References

[1] Bagale R., Acharya S., Gupta A., Chaudhary P., Chaudhary G.P., Pandey J. (2022). Antibacterial and antioxidant activities of *Prinsepia utilis* Royle leaf and seed extracts. J. Trop. Med..

[2] Editorial Committee of Flora of China, Chinese Academy of Sciences (1986). Flora Reipublicae Popularis Sinicae.

[3] Zhao Y.Q., Zhao Y., Yang L.X. (2023). Ethnobotanical study on *Prinsepia utilis* and its skin care-related traditional knowledge. Guihaia.

[4] Lan M. (1975). Diannan Bencao.

[5] (1976). The Wealth of India: A Dictionary of Indian Raw Materials and Industrial Products.

[6] Guan B., Peng C.C., Zeng Q., Cheng X.R., Yan S.K., Jin H.Z., Zhang W.D. (2013). Cytotoxic pentacyclic triterpenoids from *Prinsepia utilis*. Planta Med..

[7] Chauhan K., Bhalla P., Chitme H.R., Varshney V.K. (2024). Exploring the therapeutic potential of *Prinsepia utilis* Royle seed oil: A comprehensive study on chemical composition, physicochemical properties, anti-inflammatory, and analgesic activities. J. Ethnopharmacol..

[8] Li C., He R., Yin X., Inta A., Ali M., Gao L., Yao R., Yang L. (2026). Bioactive compounds and related food-medicine homology potential of *Prinsepia utilis* seed oil. Molecules.

[9] Chauhan K., Tripathi Y.C., Varshney V.K. (2023). *Prinsepia utilis* Royle: A review on its traditional uses, phytochemistry, and biological activities. Phytochem. Lett..

[10] Guan B., Li T., Xu X.K., Zhang X.F., Wei P.L., Peng C.C., Fu J.J., Zeng Q., Cheng X.R., Zhang S.D. (2014). γ-Hydroxynitrile glucosides from the seeds of *Prinsepia utilis*. Phytochemistry.

[11] Zhang X.J. (2019). Chemical Constituents of the Fruits of *Prinsepia utilis*. Master’s Thesis.

[12] Guan B., Peng C.C., Wang C.H., Jin H.Z., Zhang W.D. (2014). Chemical constituents from the aerial parts of *Prinsepia utilis*. Chem. Nat. Compd..

[13] Wang B., Wang F., Gao H. (2022). *Prinsepia utilis* Royle oil extract improve skin barrier on reconstructed skin model. Int. J. Nurs. Health Care Res..

[14] Tu Y., An R., Gu H., Li N., Yan H., Liu H.Y., He L. (2024). The water extracts from the oil cakes of *Prinsepia utilis* repair the epidermal barrier via up-regulating corneocyte envelope proteins, lipid synthases, and tight junction proteins. J. Ethnopharmacol..

[15] Zheng Y., Zhao L., Yi J. (2022). Phytochemical characterization and antioxidant and enzyme inhibitory activities of different parts of *Prinsepia utilis* Royle. J. Food Qual..

[16] Liu J., Qu L., Wang F., Mei Z., Wu X., Wang B., Liu H., He L. (2024). A study on the anti-senescent effects of flavones derived from *Prinsepia utilis* Royle seed residue. J. Ethnopharmacol..

[17] Gupta R., Goyal R., Bhattacharya S., Dhar K.L. (2015). Antioxidative in vitro and antiosteoporotic activities of *Prinsepia utilis* Royle in female rats. Eur. J. Integr. Med..

[18] Guo Y., Peng X., Liu F., Zhang Q., Ding L., Li G., Qiu F. (2024). Potential of natural products in inflammation: Biological activities, structure-activity relationships, and mechanistic targets. Arch. Pharm. Res..

[19] Shen W., Li S.Y., Pan Y.Q., Liu H., Dong X.W., Zhang X.Q., Ye W.C., Hu X.L., Wang H. (2023). *Prinsepia utilis* Royle leaf extract: Ameliorative effects on allergic inflammation and skin lesions in allergic contact dermatitis and polyphenolic profiling through UPLC-MS/MS coupled to chemometric analysis. J. Ethnopharmacol..

[20] Chen L., Wu M., Jiang S., Zhang Y., Li R., Lu Y., Liu L., Wu G., Liu Y., Xie L. (2019). Skin toxicity assessment of silver nanoparticles in a 3D epidermal model compared to 2D keratinocytes. Int. J. Nanomed..

[21] Rothenbücher T.S.P., Ledin J., Gibbs D., Engqvist H., Persson C., Hulsart-Billström G. (2019). Zebrafish embryo as a replacement model for initial biocompatibility studies of biomaterials and drug delivery systems. Acta Biomater..

[22] Sumner L.W., Amberg A., Barrett D., Beale M.H., Beger R., Daykin C.A., Fan T.W., Fiehn O., Goodacre R., Griffin J.L. (2007). Proposed minimum reporting standards for chemical analysis: Chemical Analysis Working Group (CAWG), Metabolomics Standards Initiative (MSI). Metabolomics.

[23] Merecz-Sadowska A., Sadowski A., Zielińska-Bliźniewska H., Zajdel K., Zajdel R. (2025). Network pharmacology as a tool to investigate the antioxidant and anti-inflammatory potential of plant secondary metabolites-A review and perspectives. Int. J. Mol. Sci..

[24] Riaz M., Khalid R., Afzal M., Anjum F., Fatima H., Zia S., Rasool G., Egbuna C., Mtewa A.G., Uche C.Z. (2023). Phytobioactive compounds as therapeutic agents for human diseases: A review. Food Sci. Nutr..

[25] Manivannan H.P., Veeraraghavan V.P., Francis A.P. (2025). Prediction of multi-targeting pharmacological activity of bioactive compounds from medicinal plants against hepatocellular carcinoma through advanced network pharmacology and bioinformatics-based investigation. Appl. Biochem. Biotechnol..

[26] Bai X., Guo Y.R., Zhao Z.M., Li X.Y., Dai D.Q., Zhang J.K., Li Y.S., Zhang C.D. (2025). Macrophage polarization in cancer and beyond: From inflammatory signaling pathways to potential therapeutic strategies. Cancer Lett..

[27] Veerasubramanian P.K., Wynn T.A., Quan J., Karlsson F.J. (2024). Targeting TNF/TNFR superfamilies in immune-mediated inflammatory diseases. J. Exp. Med..

[28] Ma Q., Hao S., Hong W., Tergaonkar V., Sethi G., Tian Y., Duan C. (2024). Versatile function of NF-κB in inflammation and cancer. Exp. Hematol. Oncol..

[29] Huang J., Gao J., Chen Q. (2025). An interpretable deep learning and molecular docking framework for repurposing existing drugs as inhibitors of SARS-CoV-2 main protease. Molecules.

[30] Salentin S., Schreiber S., Haupt V.J., Adasme M.F., Schroeder M. (2015). PLIP: Fully automated protein-ligand interaction profiler. Nucleic Acids Res..

[31] He R., Liang Z., Liu J., Wei J., Sun Z., Lv J., Wang Q., Yao M., Li G., Wei H. (2026). Molecular mechanisms of Shenhong Tongluo Formula against myocardial ischemia-reperfusion injury: Insights from RNA sequencing, bioinformatics, reverse network pharmacology, molecular docking, and molecular dynamics simulations combined with experimental studies. Phytomedicine.

[32] De Oliveira S., Reyes-Aldasoro C.C., Candel S., Renshaw S.A., Mulero V., Calado A. (2013). Cxcl8 (IL-8) mediates neutrophil recruitment and behavior in the zebrafish inflammatory response. J. Immunol..

[33] Guangzhou Development District Huangpu Cosmetics Industry Association (2023). Soothing Efficacy of Cosmetics-Zebrafish Embryo Neutrophil Test Method.

[34] Watkins S.C., Maniar S., Mosher M., Roman B.L., Tsang M., St Croix C.M. (2012). High-resolution imaging of vascular function in zebrafish. PLoS ONE.

[35] Sun J., Jiang Y., Fu J., He L., Guo X., Ye H., Yin C., Li H., Jiang H. (2024). Beneficial effects of epigallocatechin gallate in preventing skin photoaging: A review. Molecules.

[36] Bai D., Wang Z., Xiao Y., Liu T., Pu Y., Sun H., Wang M., Guo C., Zhang J. (2024). Transdermal delivery of elastin peptide assisted by betaine-based deep eutectic solvent to ameliorate skin photoaging. Biomater. Adv..

[37] Xie A., Wu J., Huang Q. (2025). Total *Panax notoginseng* saponins repair the epidermal barrier by regulating a multi-pathway network: Insights from an integrative RHE model and multi-omics study. Int. J. Mol. Sci..

[38] Ji K.Y., Kim K.M., Kim Y.H., Im A.R., Lee J.Y., Park B., Na M., Chae S. (2019). The enhancing immune response and anti-inflammatory effects of *Anemarrhena asphodeloides* extract in RAW 264.7 cells. Phytomedicine.

[39] Zhou Z., Luo M., Zhang H., Yin Y., Cai Y., Zhu Z.J. (2022). Metabolite annotation from knowns to unknowns through knowledge-guided multi-layer metabolic networking. Nat. Commun..

[40] Wang L., Wang C., Jiang L., Hu H., Wu Y., Li Y., Sun P. (2025). Efficacy of Encorelane in enhancing barrier function and reducing aging signs in sensitive skin. J. Cosmet. Dermatol..

[41] Henry K.M., Loynes C.A., Whyte M.K., Renshaw S.A. (2013). Zebrafish as a model for the study of neutrophil biology. J. Leukoc. Biol..

[42] Sugden W.W., Meissner R., Aegerter-Wilmsen T., Tsaryk R., Leonard E.V., Bussmann J., Hamm M.J., Herzog W., Jin Y., Jakobsson L. (2017). Endoglin controls blood vessel diameter through endothelial cell shape changes in response to haemodynamic cues. Nat. Cell Biol..

