# Supplementary material for: Integrated Metabolomics and Multilevel Validation of the Anti-Inflammatory and Skin-Soothing Effects of Bioactive Components from Prinsepia utilis Seeds
Source: Int J Mol Sci. 2026 Sep 7;27(17):7951. doi: 10.3390/ijms27177951 (PMC13566757; doi:10.3390/ijms27177951)
Supplement: Supplementary file 1 [file ijms-27-07951-s001.zip › Supplementary File S1.pdf]

1. TIC Plots of Samples from Non-Targeted Metabolomics Experiments

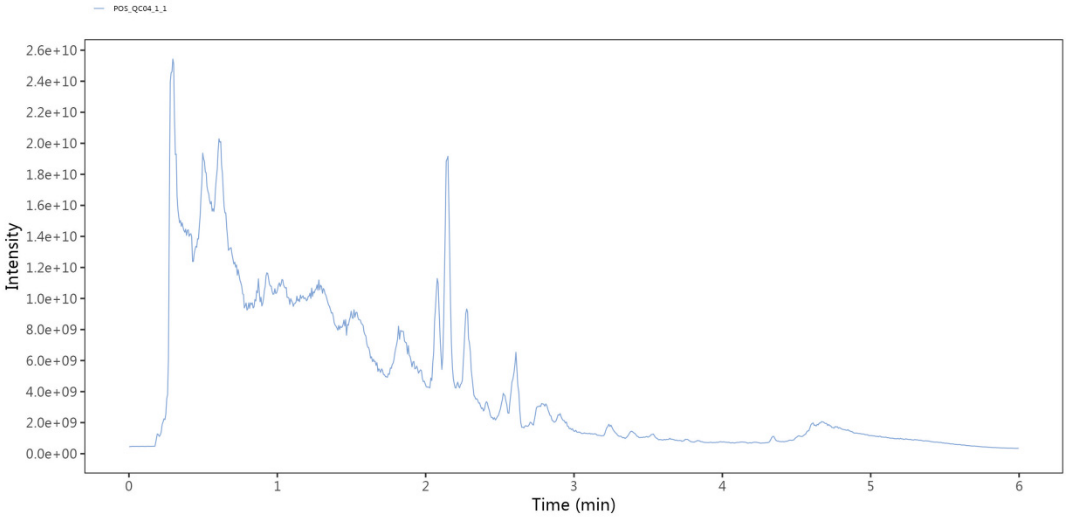

Figure S1. TIC plot of a sample in positive-ion mode in a polar metabolite system

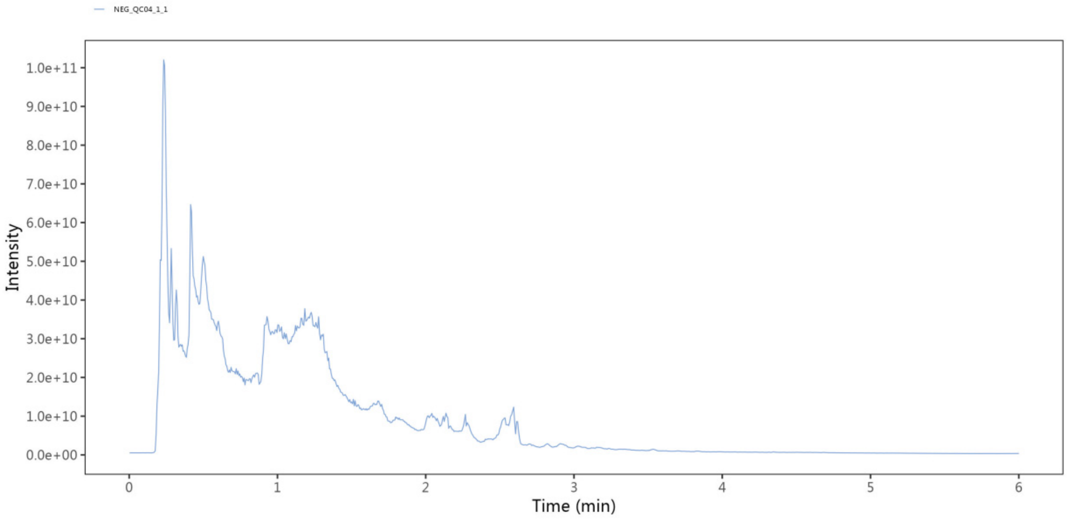

Figure S2. TIC plot of the anionic mode for sample analysis in a polar metabolite system

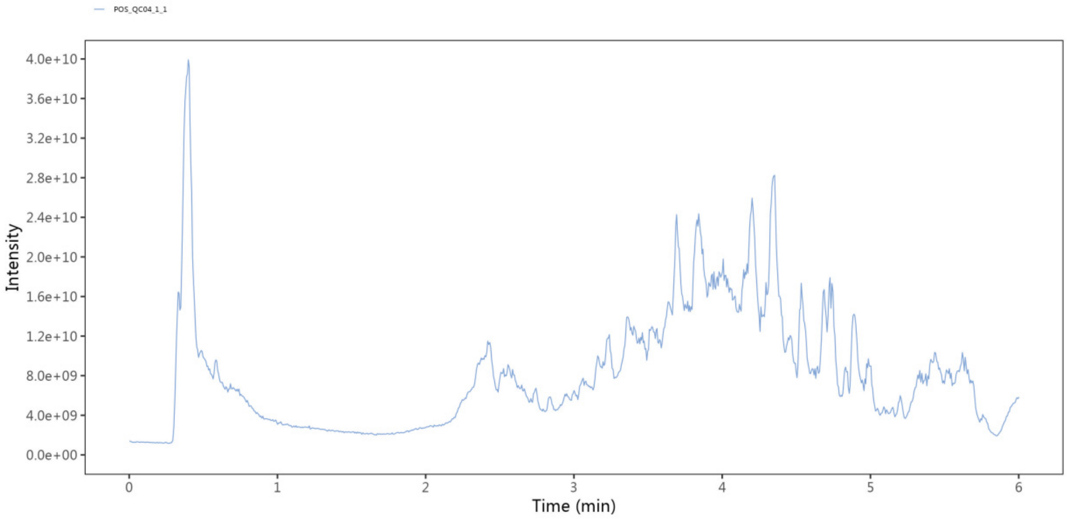

Figure S3. TIC plot of sample cations in a non-polar metabolite system

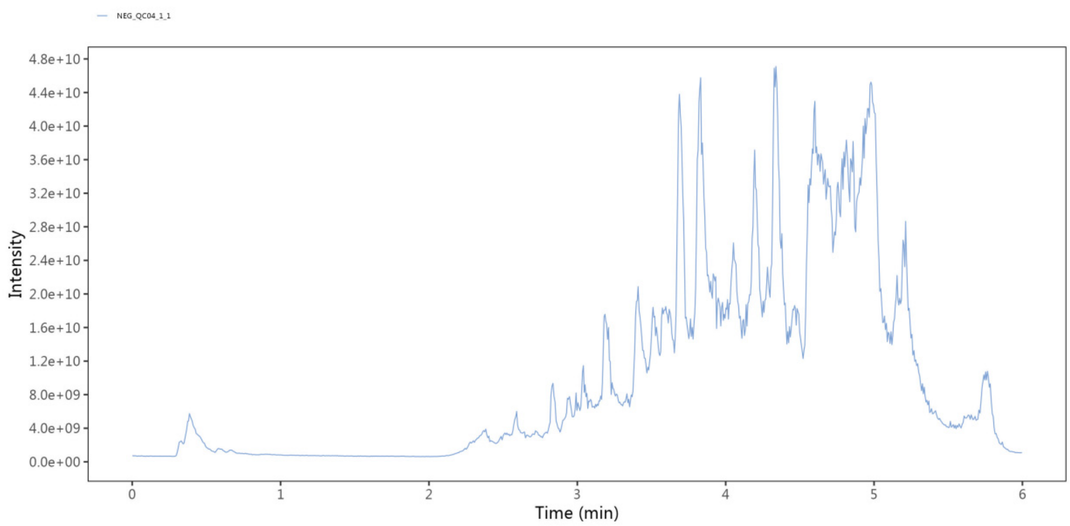

Figure S4. IC plot of the anionic mode for samples in a non-polar metabolite system

2. Quality Control

2.1 RTQC Mixed Standard Retention Time Stability

As the instrument operates, the retention times of metabolites may fluctuate; therefore, it is necessary to monitor the retention times of metabolites. RTQC is a mixture of multiple metabolite standards. Multiple analyses of the RTQC mixture are performed at different time intervals over a 24-hour period. Thus, the smaller the variation in the retention times of the RTQC mixture (which should be <30 s), the more stable the system and the higher the data quality. As can be seen from the figure, the data quality in this experiment is very high.

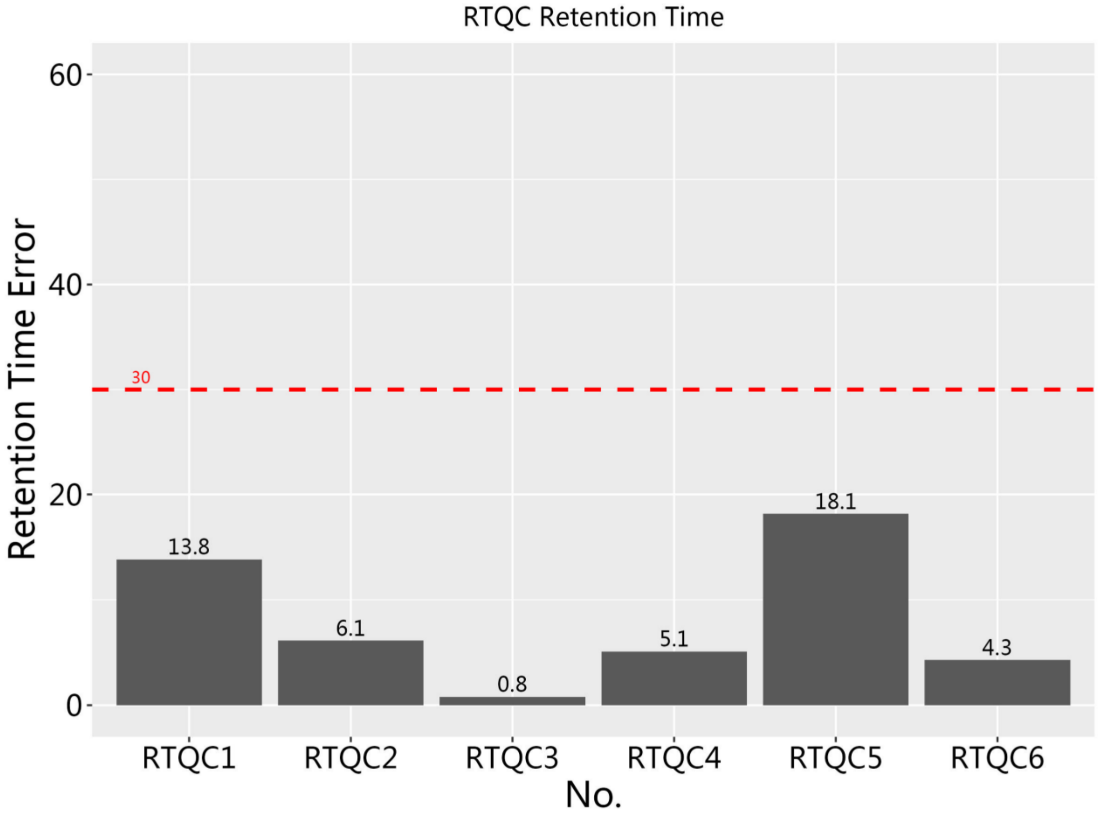

Figure S5. Analysis of retention time variability in RTQC mixed standards

2.2 RTQC Mixed Standard Retention Time Correction Scatter Plot

After calibrating the retention time model using RTQC mixed standards and plotting the calibrated scatter plot, a higher R^2 value (which should be at least >0.9) and a slope in the range of 0.8 to 1.2 indicate that the system is more stable and the data quality is higher. As can be seen from the graph, the data quality in this experiment is very high.

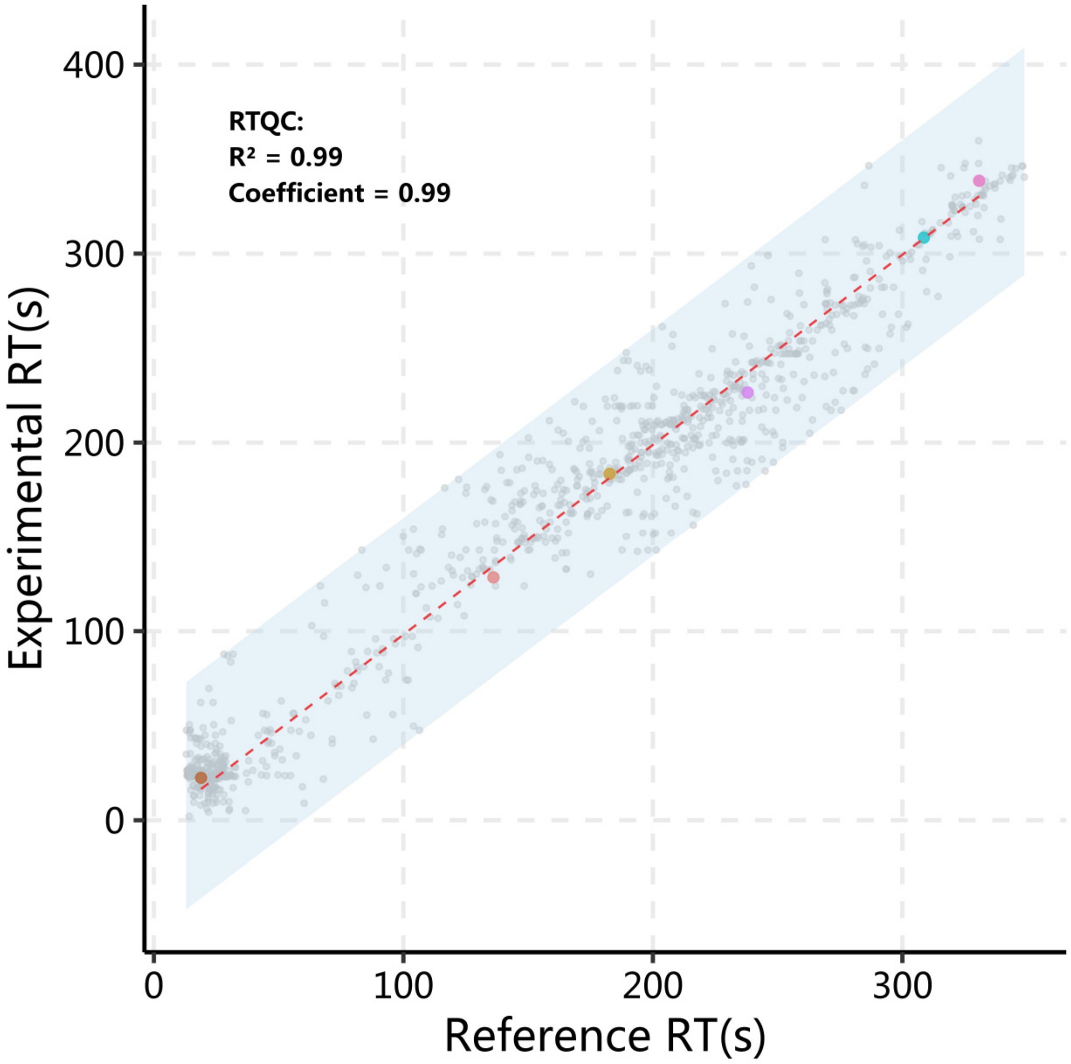

Figure S6. Scatter plot of retention time correction for RTQC mixture standards. The gray data points represent Level 1 substances under analysis, while the colored data points represent RTQC mixture standards.
