# Supplementary material for: Integrated Metabolomics and Multilevel Validation of the Anti-Inflammatory and Skin-Soothing Effects of Bioactive Components from Prinsepia utilis Seeds
Source: Int J Mol Sci. 2026 Sep 7;27(17):7951. doi: 10.3390/ijms27177951 (PMC13566757; doi:10.3390/ijms27177951)
Supplement: Supplementary file 1 [file ijms-27-07951-s001.zip › Supplementary File S2/Madecassic acid.pdf]

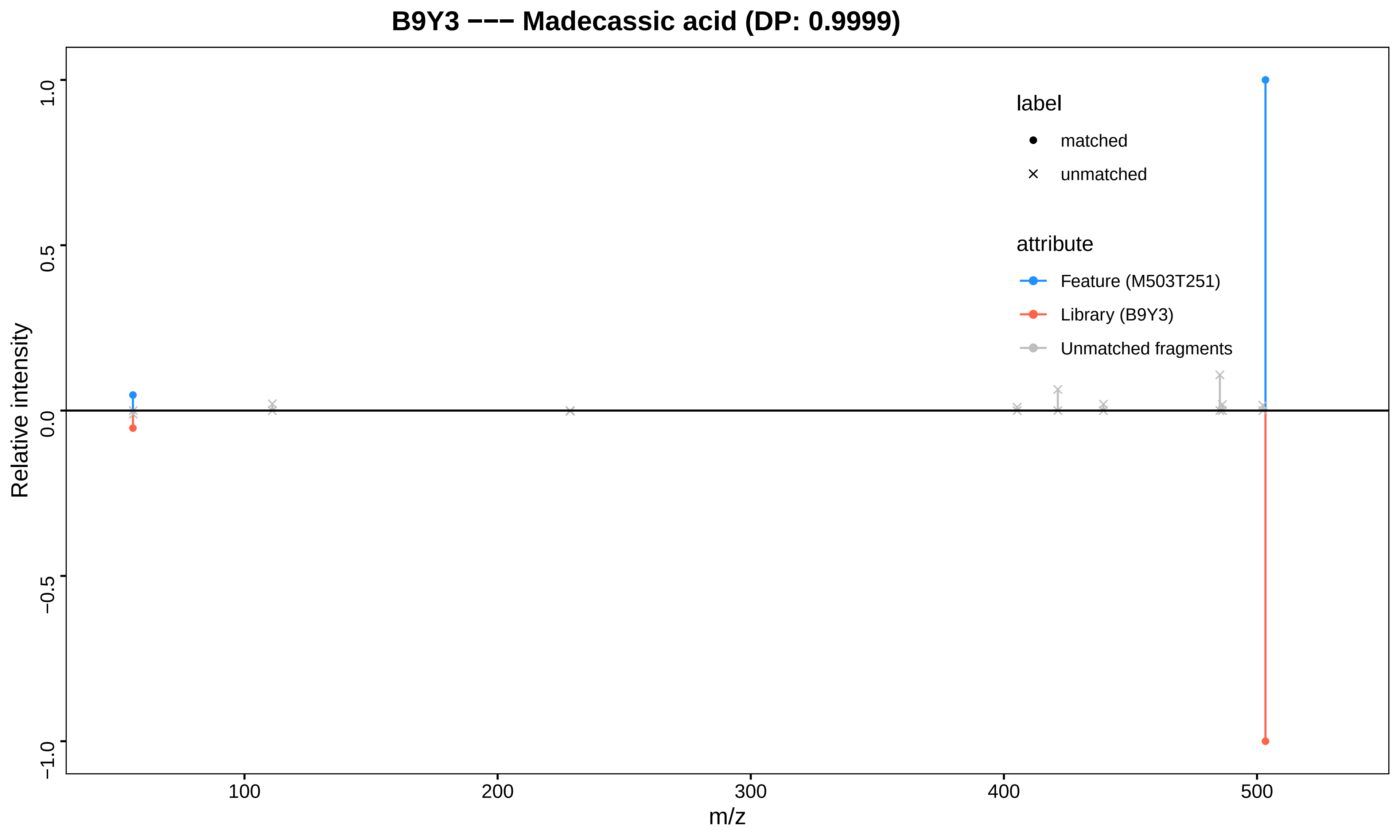
B9Y3 --- Madecassic acid (DP: 0.9999)
Relative intensity
m/z
1.0
0.5
0.0
−0.5
−1.0
100
200
300
400
500
label
matched
unmatched
attribute
Feature (M503T251)
Library (B9Y3)
Unmatched fragments
