# Supplementary material for: Integrated Metabolomics and Multilevel Validation of the Anti-Inflammatory and Skin-Soothing Effects of Bioactive Components from Prinsepia utilis Seeds
Source: Int J Mol Sci. 2026 Sep 7;27(17):7951. doi: 10.3390/ijms27177951 (PMC13566757; doi:10.3390/ijms27177951)

C0L8 --- Polygalacic acid (DP: 0.9825)

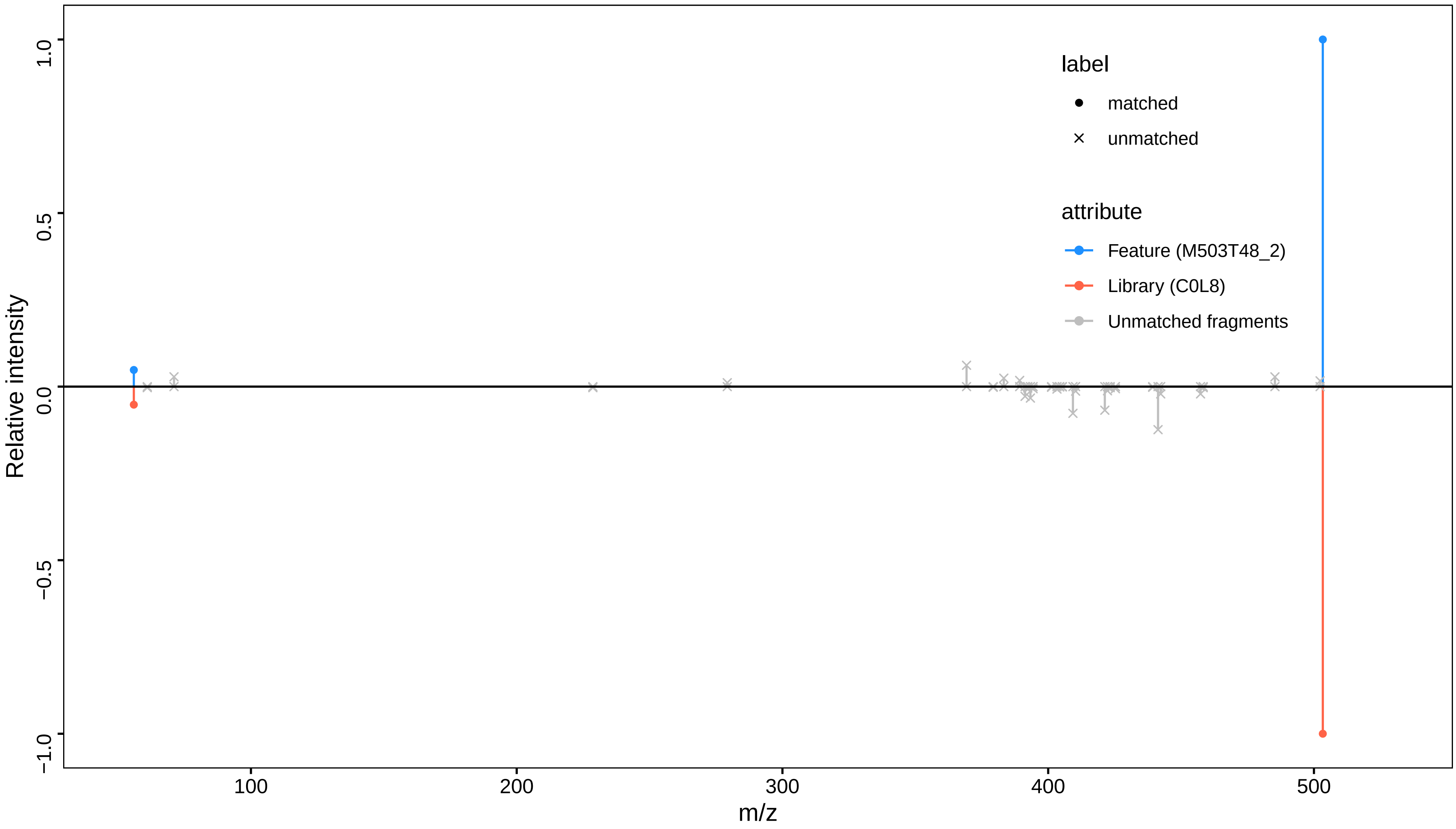

Supplement: Supplementary file 1 [file ijms-27-07951-s001.zip › Supplementary File S2/Polygalacic acid.pdf]
