# Supplementary material for: Integrated Metabolomics and Multilevel Validation of the Anti-Inflammatory and Skin-Soothing Effects of Bioactive Components from Prinsepia utilis Seeds
Source: Int J Mol Sci. 2026 Sep 7;27(17):7951. doi: 10.3390/ijms27177951 (PMC13566757; doi:10.3390/ijms27177951)
Supplement: Supplementary file 1 [file ijms-27-07951-s001.zip › Supplementary File S3.pdf]

3 Reproducibility Assessment of Molecular Dynamics Simulations

To verify the reproducibility of the results, two mutually independent molecular dynamics simulations were performed on the same system. Both simulations used identical topology and initial coordinates, with newly regenerated initial velocities (random number seeds of −50,757,753 and −285,376,533, respectively), and were run independently. Each was carried out in GROMACS 2020.6 with a 2 fs time step for 5×10^7 steps (100 ns) of sampling; the system contained 69,489 atoms. Electrostatics were treated with PME (rcoulomb = rvdw = 1.0 nm), and temperature and pressure were controlled using the V-rescale and Berendsen methods, respectively, with a reference temperature of 300 K and a reference pressure of 1 bar. The potential energies of the two trajectories diverged chaotically within 20 ps, confirming that they constitute genuinely independent replicate trajectories; both runs completed to their full length, with a constraint RMSD (Constr. rmsd) of zero throughout and no LINCS warnings or signs of numerical instability. Statistics were computed over the final 50 ns of each trajectory, after the system had fully equilibrated (block averaging, n = 10). Between the two trajectories, the differences in potential energy (−894,466 vs. −894,473 kJ/mol), total energy (−717,582 vs. −717,579 kJ/mol), temperature (299.998 vs. 300.016 K), pressure (2.65 vs. 0.89 bar), and short-range electrostatic and van der Waals energies were all far smaller than their respective thermal fluctuations (maximum deviation of only 0.15σ), and the box edge length also remained consistent (8.85852 vs. 8.85838 nm). The probability density distributions of potential energy, temperature, and pressure were all unimodal and approximately Gaussian, with overlap coefficients between the two trajectories of 0.956, 0.947, and 0.950, respectively — a level indicating that the distributions are statistically indistinguishable. Taken together, the two independent trajectories show a high degree of thermodynamic consistency, indicating that the simulation results are well reproducible.

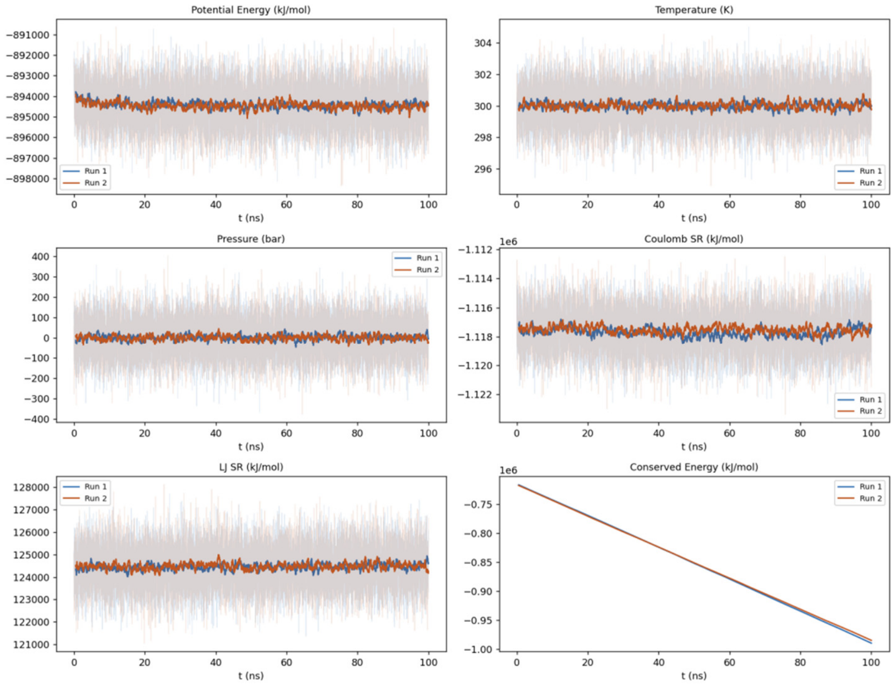

Figure S7. Time series of six thermodynamic quantities over 100 ns. Light thin lines show the raw frame-by-frame data (one frame output every 10 ps, 10,001 frames in total), and dark thick lines show the 500 ps moving average. Blue represents Run 1 and orange represents Run 2.

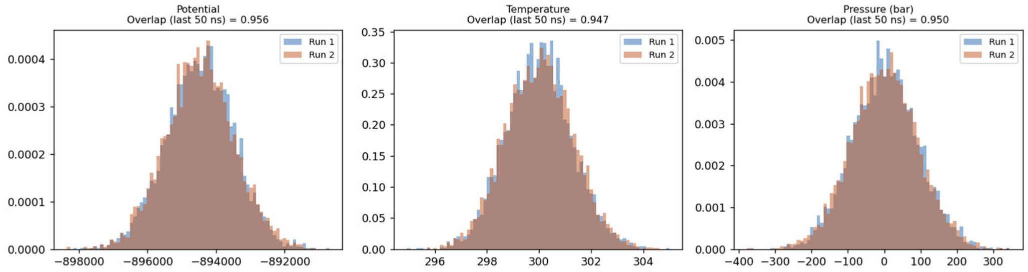

Figure S8. Normalized probability density histograms of the instantaneous potential energy, temperature, and pressure values, computed from the final 50 ns of data (after the system had fully equilibrated). The overlap coefficient between the two trajectories' distributions is given in each panel title.
