# Supplementary material for: Integrated Metabolomics and Multilevel Validation of the Anti-Inflammatory and Skin-Soothing Effects of Bioactive Components from Prinsepia utilis Seeds
Source: Int J Mol Sci. 2026 Sep 7;27(17):7951. doi: 10.3390/ijms27177951 (PMC13566757; doi:10.3390/ijms27177951)
Supplement: Supplementary file 1 [file ijms-27-07951-s001.zip › Supplementary Table S3.pdf]

Supplementary Table S3. Grid-box parameters used for AutoDock Vina molecular-docking simulations. *Note*: Docking was performed with AutoDock Vina; exhaustiveness = 32, num_modes = 9. For each receptor–ligand complex, three independent docking runs were executed, and the pose with the lowest predicted binding energy was selected for further analysis. For targets 3QKK, 5NZZ and 8PT1, native-ligand re-docking was performed to assess protocol performance against a 2.0 Å RMSD acceptance threshold. For target 3V3V (no co-crystallized ligand), re-docking validation relied on the homologous structure PDB 1UKI. Å, angstrom.

Target protein (PDB ID)	Grid-box source	Center coordinates (x, y, z) (Å)	Grid-box dimensions (x × y × z) (Å)
3QKK (AKT1)	Centered on co-crystallized ligand	28.78, 0.48, 10.91	25.64 × 30.50 × 24.08
5NZZ (MAPK14)	Centered on co-crystallized ligand	60.85, 91.94, 27.21	21.72 × 26.24 × 28.65
3V3V (MAPK8)	Centered on co-crystallized ligand	20.78, 41.94, 8.90	75.83 × 73.92 × 65.81
8PT1 (MAPK1)	Centered on co-crystallized ligand	−22.57, −32.73, 21.70	23.07 × 24.54 × 23.29
